# Unraveling the relative role of light and water competition between lianas and trees in tropical forests: A vegetation model analysis

**DOI:** 10.1111/1365-2745.13540

**Published:** 2020-11-29

**Authors:** Félicien Meunier, Hans Verbeeck, Betsy Cowdery, Stefan A. Schnitzer, Chris M. Smith‐Martin, Jennifer S. Powers, Xiangtao Xu, Martijn Slot, Hannes P. T. De Deurwaerder, Matteo Detto, Damien Bonal, Marcos Longo, Louis S. Santiago, Michael Dietze

**Affiliations:** ^1^ Computational and Applied Vegetation Ecology Department of Environment Ghent University Ghent Belgium; ^2^ Department of Earth and Environment Boston University Boston MA USA; ^3^ Smithsonian Tropical Research Institute Apartado Panama; ^4^ Department of Biological Sciences Marquette University Milwaukee WI USA; ^5^ Department of Ecology, Evolution and Evolutionary Biology Columbia University New York NY USA; ^6^ Department of Ecology, Evolution, and Behavior University of Minnesota St. Paul MN USA; ^7^ Department of Ecology and Evolutionary Biology Cornell University Ithaca NY USA; ^8^ Department of Ecology and Evolutionary Biology Princeton University Princeton NJ USA; ^9^ Université de Lorraine AgroParisTech INRAE UMR Silva Nancy France; ^10^ Jet Propulsion Laboratory California Institute of Technology Pasadena CA USA; ^11^ Department of Botany and Plant Sciences University of California Riverside CA USA

**Keywords:** competition for resources, dynamic global vegetation model, ecosystem demography model, lianas, PEcAn, plant–plant interactions, uncertainty analysis

## Abstract

1. Despite their low contribution to forest carbon stocks, lianas (woody vines) play an important role in the carbon dynamics of tropical forests. As structural parasites, they hinder tree survival, growth and fecundity; hence, they negatively impact net ecosystem productivity and long‐term carbon sequestration.
2. Competition (for water and light) drives various forest processes and depends on the local abundance of resources over time. However, evaluating the relative role of resource availability on the interactions between lianas and trees from empirical observations is particularly challenging. Previous approaches have used labour‐intensive and ecosystem‐scale manipulation experiments, which are infeasible in most situations.
3. We propose to circumvent this challenge by evaluating the uncertainty of water and light capture processes of a process‐based vegetation model (ED2) including the liana growth form. We further developed the liana plant functional type in ED2 to mechanistically simulate water uptake and transport from roots to leaves, and start the model from prescribed initial conditions. We then used the PEcAn bioinformatics platform to constrain liana parameters and run uncertainty analyses.
4. Baseline runs successfully reproduced ecosystem gas exchange fluxes (gross primary productivity and latent heat) and forest structural features (leaf area index, aboveground biomass) in two sites (Barro Colorado Island, Panama and Paracou, French Guiana) characterized by different rainfall regimes and levels of liana abundance.
5. Model uncertainty analyses revealed that water limitation was the factor driving the competition between trees and lianas at the drier site (BCI), and during the relatively short dry season of the wetter site (Paracou). In young patches, light competition dominated in Paracou but alternated with water competition between the wet and the dry season on BCI according to the model simulations.
6. The modelling workflow also identified key liana traits (photosynthetic quantum efficiency, stomatal regulation parameters, allometric relationships) and processes (water use, respiration, climbing) driving the model uncertainty. They should be considered as priorities for future data acquisition and model development to improve predictions of the carbon dynamics of liana‐infested forests.
7. *Synthesis*. Competition for water plays a larger role in the interaction between lianas and trees than previously hypothesized, as demonstrated by simulations from a process‐based vegetation model.

## Graphical abstract

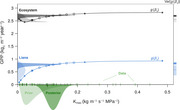

## 1 INTRODUCTION

The terrestrial biosphere is a critical component of the Earth system, responsible for the uptake of up to 30% of anthropogenic carbon emissions (Le Quéré et al., 2018). Globally, forests hold more than 80% of the terrestrial above‐ground carbon (Sedjo, 1993), about 50% of which can be found in tropical ecosystems (Pan et al., 2011). Furthermore, tropical forests account for about one‐third of terrestrial photosynthesis (Beer et al., 2010), and thus play a key role in global carbon dynamics (Wieder et al., 2015).

Lianas are woody vines which are especially abundant in tropical forests (Schnitzer & Bongers, 2011) where they comprise up to 40% of all woody stems and substantially contribute to ecosystem leaf (Schnitzer, 2002) and root (Collins et al., 2016; Smith‐Martin et al., 2019) biomass. However, their comprehensive contribution to the global carbon cycle remains poorly understood (Schnitzer, 2018).

A better understanding of the role of lianas is urgently needed as current estimates of the carbon balance of tropical ecosystems are highly uncertain (Avitabile et al., 2016; Pan et al., 2011). The widespread increase in liana abundance observed in the Neotropics (Phillips, 2002) might be among one of the multiple causes of the long‐term transition of the tropical forests from carbon sinks to net sources (Baccini et al., 2017), after decades of carbon sink strength decline (Hubau et al., 2020).

As lianas allocate less carbon to woody biomass compared to trees, they are poor contributors to long‐term forest carbon storage (van der Heijden et al., 2013) and strong competitors for resources (Alvarez‐Cansino et al., 2015; Schnitzer et al., 2005). Trees are negatively impacted by the interactions with lianas in many different ways: reduced growth (Schnitzer & Carson, 2010), increased mortality (Ingwell et al., 2010) and increased turnover (Durán & Gianoli, 2013). At the ecosystem level, a liana removal experiment in Panama revealed that tree competition with lianas was responsible for a reduction of 76% in the forest net above‐ground biomass accumulation (van der Heijden et al., 2015). Furthermore, many liana species can thrive in degraded and early successional forests, where they could slow forest regeneration and hence further strengthen the negative impact of forest disturbance on the long term.

As of today, it is unclear what (if any) mechanism dominates the competition between lianas and trees. The most limiting resource for which plant communities compete varies depending on forest site (Schnitzer, 2005), stand age (Barry et al., 2015) and season (Alvarez‐Cansino et al., 2015). Yet, light is often thought to be the main limiting factor for plant growth and development in very dense closed‐canopy ecosystems (Bongers and Sterck, 1998; Poorter et al., 2003). However, liana‐tree competition was driven by below‐ground resource acquisition (water and nutrients) in at least one tropical site of Ivory Coast (Schnitzer et al., 2005). Furthermore, water seems to play a key role in the interactions between lianas and trees as liana density is negatively correlated with mean annual precipitation and positively correlated with dry season length and site seasonality (DeWalt, 2010). Experimentally determining the relative magnitude of the different competition strengths is challenging as it requires establishing replicated manipulated field experiments, followed over time (Schnitzer et al., 2016). Process‐based vegetation models therefore have a key role to play in disentangling the different forms of competition between growth forms across sites.

Vegetation models are numerical tools that track pools and fluxes of carbon, water and energy in ecosystems. They have been routinely used for projecting ecosystem dynamics under contrasting climatic and land use scenarios (Dietze & Latimer, 2011). Despite their relevance in tropical forest dynamics, lianas have been largely ignored by dynamic vegetation models (Verbeeck & Kearsley, 2015). Recently, di Porcia e Brugnera et al. (2019) implemented a mechanistic representation of lianas into the Ecosystem Demography model (ED2) that captured the changes in net forest productivity and carbon storage caused by different levels of liana infestation, paving the road towards investigating competition between lianas and trees in silico.

Yet, di Porcia e Brugnera et al. (2019) could not identify whether competition for light or water was responsible for the reduction in ecosystem carbon storage under high liana infestation. Indeed, the original liana plant functional type (PFT) in ED2 did not include a mechanistic representation of plant water uptake and transport and was therefore limited in its potential to disentangle above‐ and below‐ground competition between lianas and trees. As a consequence, no clear signature emerged from the model simulations for sites with different hydrological drivers while liana abundance is expected to be sensitive to rainfall regime (Schnitzer & Bongers, 2011). Locally observed drought stress episodes (Alvarez‐Cansino et al., 2015) were also not reproduced by model runs.

To accurately simulate competition for resources between lianas and trees, vegetation models need to comprehensively integrate the functional differences between the two growth forms. Numerous in situ studies have indeed revealed functional and structural differences in leaf‐level gas exchange (Slot & Winter, 2017; Slot et al., 2013), hydraulic properties (De Guzman et al., 2016), rooting depth (De Deurwaerder et al., 2018; Smith‐Martin et al., 2019), root and stem vessel diameters (Ewers et al., 1997; Gartner et al., 1990), or leaf properties and allocation (Wyka et al., 2013). Those contrasts in the hydraulic architecture and functioning of lianas and trees need to be accounted for in vegetation models to determine the exact role and impact of lianas in the forest biogeochemical cycles. This was not the case in the previous model version in which several liana trait values were directly copy pasted from the list of ED2 pioneer tree parameters.

The objective of this study is to estimate the relative contribution of below‐ and above‐ground competition between lianas and trees in order to better predict the dynamics of tropical forests as affected by lianas. In particular, we aim to (a) determine how lianas contribute to tropical forest ecosystem fluxes and plant community competition, (b) identify the liana physiological/ecological parameters that contribute the most to liana‐tree competition and (c) assess the relative strengths of above‐ and below‐ground competition between lianas and trees over time and across sites and forest stand ages.

To do so, we first updated the ED2 liana plant functional type to include the plant hydraulics module recently implemented in ED2 (Xu et al., 2016). We then used the Predictive Ecosystem Analyser (PEcAn, LeBauer et al., 2013) to (a) exhaustively parameterize the liana PFT according to the most recent available observational data and (b) run an uncertainty analysis in order to identify where and when light (or water) was the most limiting resource in two sites (Paracou, French Guiana and Barro Colorado Island [BCI], Panama). These two sites are relatively wet (yearly rainfall of about 3,100 and 2,650 mm, respectively) but differ in the length and intensity of their dry season. In particular, we wanted to investigate if the relatively short and weak dry season in Paracou was sufficient to trigger a strong water competition between lianas and trees. We hypothesized that the quest for water would drive liana‐tree competition on BCI where the dry season is longer and stronger. Contrastingly, under the wet conditions prevailing in Paracou, we expected competition to be primarily for light. In both sites, we assumed that young patch dynamics (where light is abundant, and root systems not fully developed) would be mainly driven by water competition. The comprehensive liana PFT meta‐analysis allowed us to better constrain the model parameters, update the original implementation of di Porcia e Brugnera et al. (2019) and estimate the reduction of parameter uncertainty gained thanks to such a literature review.

## 2 MATERIALS AND METHODS

### 2.1 Model description

#### 2.1.1 The ecosystem demography model

The Ecosystem Demography model version 2 (ED2) is a terrestrial biosphere model that accounts for horizontal and vertical heterogeneity across the landscape as well as plant diversity (Medvigy et al., 2009). ED2 is a size‐ and age‐structured approximation of an individual vegetation model that is able to represent the stochastic nature of mortality, reproduction and dispersal processes (Longo, Knox, Medvigy, et al., 2019). ED2 simulates both the short‐term response of the ecosystem to changes in atmospheric conditions as well as the long‐term dynamics of ecosystem composition driven by resource limitations (Raczka et al., 2018), which makes it a suitable tool to investigate competition between growth forms or functional groups.

In ED2, the energy, carbon and water cycles are solved separately for each single group of plants belonging to the same functional type and sharing a similar diameter at breast height (DBH), that is, the plant cohorts (Moorcroft et al., 2001). The cohorts belong to patches, which are defined as areas of the forest with a certain age, that is, time since last disturbance. Each patch represents the collection of similar canopy gap‐sized areas within a given site (Moorcroft et al., 2001). Patch area corresponds to the relative chance of finding a forest portion sharing the same disturbance history. Plant cohorts and patches are spatially implicit: the horizontal position of each plant in a patch and the position of patches relative to one another are not simulated (Longo, Knox, Medvigy, et al., 2019). Instead, the model computes the plant density of each cohort within each patch and its dynamics.

Previous studies have demonstrated the capacity of the ED2 model to realistically simulate important aspects of carbon and water dynamics in different types of ecosystems: temperate (Medvigy & Moorcroft, 2012; Medvigy et al., 2009), boreal (Ise & Moorcroft, 2010) and tropical (Longo, Knox, Levine, et al., 2019). Importantly, ED2 could reproduce reductions in above‐ground biomass of Amazon forests subjected to drought experiments (Powell et al., 2013), capture multiple benchmarks (e.g. mortality rates, above‐ground biomass stocks) on Barro Colorado Island, Panama (Powell, Kueppers, et al., 2017), and represent leaf and biomass spatial and temporal variability in tropical dry forests (Xu et al., 2016).

#### 2.1.2 Model relevant processes and parameter description

Among other biological and physical processes, ED2 simulates soil hydrology (Walko et al., 2000), biogeochemistry (Bolker et al., 1998), leaf phenology (Botta et al., 2000), photosynthesis (Farquhar et al., 1980) and plant hydraulics (Xu et al., 2016), which all in turn impact the energy, carbon and water balances of the ecosystem. For further details about the model structure, we refer the readers to the latest model description (Longo, Knox, Medvigy, et al., 2019) as we only briefly describe a subset of the model parameters and the underlying processes relevant to this study.

#### 2.1.3 The plant functional types

Plant functional types (PFTs) reflect an ensemble of morphological, physiological and life‐history traits that mimic the plant strategy for resource acquisition and use (Fisher et al., 2010). In this study, we simulated the competition for light and water between one liana PFT and three tree (early‐, mid‐ and late‐successional tropical evergreen trees) PFTs. We used the tree PFT definitions of Longo, Knox, Medvigy, et al. (2019), in which self‐supporting plants are represented by a discrete approximation of the continuous distribution of life strategies, ranging from fast‐growing, resource‐acquisitive (early‐successional PFT) to conservative, slow‐growing (late‐successional PFT).

For this analysis, we focused on lianas and selected 32 parameters related to various aspects of their ecophysiology, competition and demography (Table 1). These specific plant parameters were chosen based on previous sensitivity and uncertainty analyses (Dietze et al., 2014; LeBauer et al., 2013; Wang et al., 2013) and the prior knowledge of their importance for lianas (di Porcia e Brugnera et al., 2019). Detailed information on processes modulated by the selected parameters is available in Appendix A. For the tropical tree PFTs, we used the same parameterization as Longo, Knox, Medvigy, et al. (2019).

**TABLE 1:** List of model parameters for the liana PFT analysed in this study alongside with their description, units and classification into organs, competition type and model processes. A more detailed description of the underlying processes that those parameters affect can be found in Appendix A

Parameter	Description	Units	Competition	Organ	Process
b1Bl	DBH‐leaf allometry intercept	kg_C_/cm^b2Bl^	Light	Leaf	Allocation
b2Bl	DBH‐leaf allometry slope	—	Light	Leaf	Allocation
b1Bs	DBH‐stem allometry intercept	kg_C_/cm^b2Bs^	—	Stem	Allocation
b2Bs	DBH‐stem allometry slope	—	—	Stem	Allocation
q	Ratio of carbon allocated to fine roots/leaves	g_C_/g_C_	Water	Root	Allocation
b1Rd	DBH‐rooting depth allometry intercept	m/m^b2Rd^	Water	Root	Allocation
b2Rd	DBH‐rooting depth allometry slope	—	Water	Root	Allocation
b1Ht	DBH‐height allometry intercept	—	Light	Stem	Allocation
b2Ht	DBH‐height allometry slope	cm^−1^	Light	Stem	Allocation
Reproduction carbon	Storage carbon allocated to recruitment	g_C_/g_C_	—	Seed	Allocation
Root beta	Fraction of root biomass below max. root depth		Water	Root	Water use
SRA	Specific root area	m^2^ kg_C_	Water	Root	Water use
*K*_max_	Maximum hydraulic conductivity of the stem	kg m^−1^ s^−1^ m^−1^	Water	Stem	Water use
*K*_exp_	Exponent for the hydraulic vulnerability curve of stem conductivity (Weibull)	—	Water	Stem	Water use
P50	Water potential at which 50% of stem conductivity is lost	m	Water	Stem	Water use
Wood capacitance	Wood hydraulic capacitance	kg kg^−1^ m^−1^	Water	Stem	Water use
Leaf capacitance	Leaf hydraulic capacitance	kg kg^−1^ m^−1^	Water	Leaf	Water use
stoma_psi_b	Water potential scaled to modify stomatal conductance under drought stress	m	Water	Leaf	Water use
stoma_psi_c	Exponent to modify stomatal conductance under drought stress	—	Water	Leaf	Water use
leaf TLP	Leaf turgor loss point	m	Water	Leaf	Water use
*V*_m0_	Maximum photosynthetic capacity at a reference temperature (15°C)	µmol_c_ m^−2^ s^−1^	Light	Leaf	Photosynthesis
Quantum efficiency	Efficiency of using PAR to fix CO_2_	—	Light	Leaf	Photosynthesis
Stomatal slope	Ball–Berry stomatal parameter	—	Light	Leaf	Photosynthesis
Root respiration	Contribution of roots to respiration	µmol_c_ kg^−1^ s^−1^	Water	Root	Respiration
Dark respiration	Rate of dark (leaf) respiration	—	Light	Leaf	Respiration
Growth respiration	Fraction of assimilated carbon to growth respiration	g_C_/g_C_	—	Entire plant	Respiration
mort_2_	Negative carbon balance mortality shape parameter	year^−1^	—	Entire plant	Mortality
mort_3_	Density‐independent (ageing) mortality	year^−1^	—	Entire plant	Mortality
Leaf turnover	Carbon cost parameter for leaf turnover	year^−1^	Light	Leaf	Tissue turnover
Root turnover	Carbon cost parameter for fine root turnover	year^−1^	Water	Root	Tissue turnover
SLA	Leaf area per leaf mass	m^2^ kg_C_	Light	Leaf	Structural
rho	Wood density	g/cm^3^	—	Stem	Structural

**FIGURE 1:**
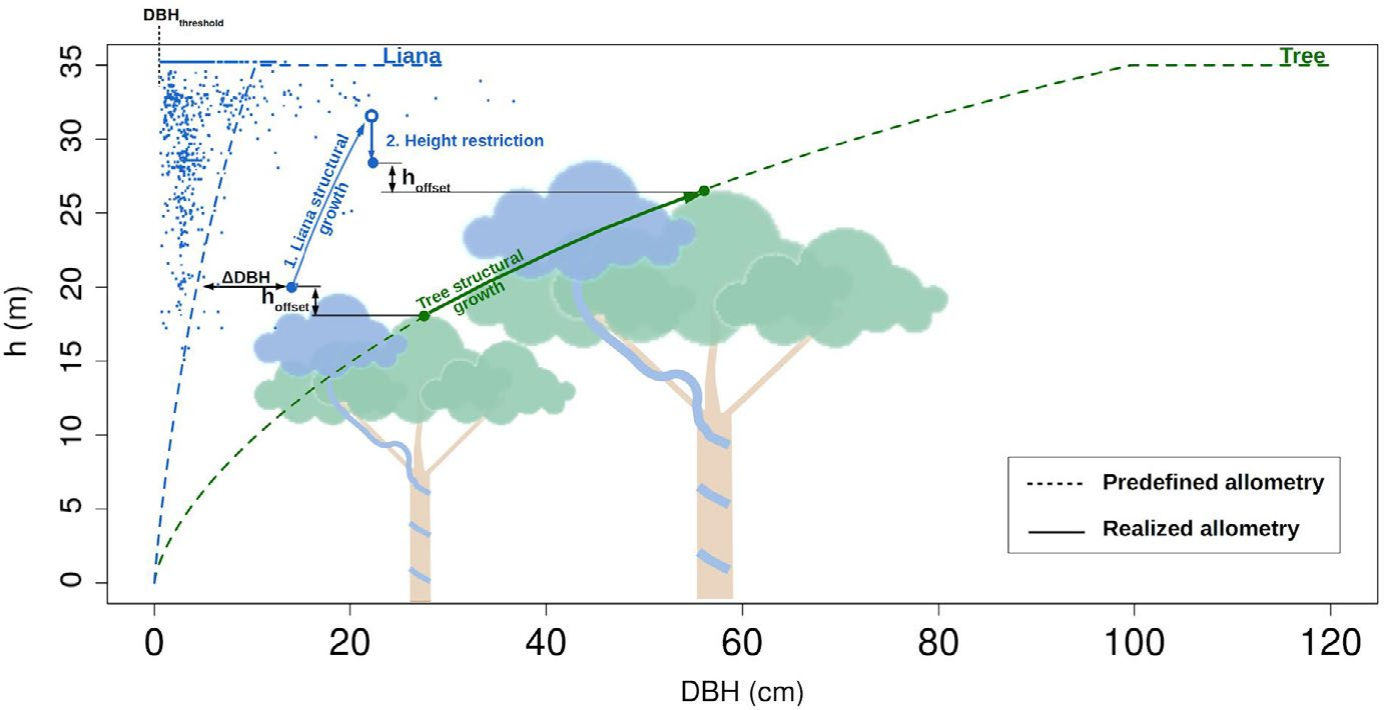
Liana model and initial forest composition. The figure illustrates the DBH‐height (*h*) allometry for both trees (dashed green line) and lianas (dashed blue line) as well as the liana initial distribution on BCI as derived from forest inventory and allometric equation (blue dots). Initially, all liana cohorts larger than DBH_threshold_ are assumed to have reached the canopy (i.e. to be slightly taller than the tallest tree within that specific forest patch). For each single liana cohort (each blue dot in the graph), an initial DBH‐offset (ΔDBH) is calculated as the DBH‐difference between the allometric equation and the actual allometric position and is used to shift the cohort DBH‐height allometric relationship. As opposed to trees, the liana growth is a two‐step process: the available carbon is spent by lianas to grow in diameter and compute a potential height which is further restricted by the tallest tree height within that patch incremented by a small offset (*h*_offset_). ΔDBH is then updated with its new value

We implemented a few important changes to the original representation of lianas in ED2 (di Porcia e Brugnera et al., 2019). First, the liana PFT was integrated in the most recent model version of ED2 that includes plant hydraulics and a process‐based description of water uptake and transport (Powell et al., 2018; Xu et al., 2016). Second, as simulations were initialized with observed inventory data rather than started from bare ground, we introduced height restrictions for liana cohorts based on the patch tree height distribution rather than the height of a tracked cohort. Liana heights were allowed to deviate from the prescribed height allometry so that large lianas can overtop the tallest tree cohort in each forest patch by no more than a small offset (Figure 1). Without the structural support of the host tree, they can indeed not grow any higher.

In the explanatory schematics of Figure 1, the forest is composed of a liana cohort overtopping a tree cohort by a fixed maximal *h*_offset_. Because of carbon allocation to growth, both tree and liana cohorts increase in diameter and hence height (tree/liana structural growth). However, the resulting liana height is larger than the updated tree cohort height and is therefore reduced to just overtop it (height restriction), which results in a deviation from the prescribed allometry. Liana initial height was similarly restricted for all lianas larger than a threshold DBH fixed to 3 cm. Details of implementation are given in Appendix B. In addition, we give an overview of the liana plant functional type functioning as well as the details of the differences with the original implementation of di Porcia e Brugnera et al. (2019) in Appendix C.

### 2.2 Model predictive uncertainty and parameter sensitivity

To quantify the model uncertainty with respect to liana‐tree competition, we used the automated workflow in PEcAn, which consists of three main steps (LeBauer et al., 2013): (a) a meta‐analysis to constrain PFT functional, physiological and morphological parameters from observational trait data, (b) a model sensitivity analysis of the selected parameters and (c) an uncertainty analysis that combines the results of the first two steps to estimate the relative importance of each parameter on the overall parametric uncertainty. In this study, we kept tree parameters constant while letting liana parameters vary. Lianas being in competition with a range of competitors (slow‐ to fast‐acquisitive tropical trees), we assumed that incorporating tree parameters in the sensitivity analysis would only increase the number of parameters without clarifying the picture.

#### 2.2.1 Meta‐analysis

First, the meta‐analysis aims to generate a posterior distributions ($\beta_{0_{p}}$) for each parameter *p* from a prior distribution and the existing trait observational data. Prior distributions represent the a priori knowledge of the model parameters and define the widest range of variation as well as the probabilistic distribution of each single trait. Liana priors were adapted from tree distributions (Dietze et al., 2014; LeBauer et al., 2013; Raczka et al., 2018) to encompass the original parameterization of the liana PFT and reflect the allegedly differences between growth forms according to ‘liana/ED2 expert’ opinion, see Table 2. Prior distributions were also chosen to generate medians close to ED2 default values, as defined in di Porcia e Brugnera et al. (2019). This allowed us to estimate the impact of the original potential mis‐parameterization of the liana PFT. Liana data were collected through an extensive literature search using Web of Science and Google scholar as search engines with a combination of ‘liana/woody vine’ and the corresponding trait or process name as keywords. All extracted data were stored in PEcAn's companion database BETYdb (LeBauer et al., 2018). Posterior distributions were then estimated using a linear mixed model further detailed in Appendix D.

**TABLE 2:** Parameter distributions for the liana PFT as used in the sensitivity analysis alongside with the prior and posterior medians and the ED2 default parameters (di Porcia e Brugnera et al., 2019). The values *a* and *b* define the constants of the prior distribution function (LeBauer et al., 2013) for each parameter analysed in this study. The sample size (*N*) is the number of mean trait observations collected for the meta‐analysis. Parameter units can be found in Table 1

Parameter	Prior (*a*, *b*)^a^	*a*	*b*	Prior median	ED2 default	Posterior median^b^	*N* ^c^	CV_p,posterior_/CV_p,prior_ ^d^
b1Bl	unif	0.005	0.15	0.0078	0.0086^f^	0.0096	462 (4)	0.07
b2Bl	unif	1.6	2.2	1.9	2^f^	1.85	462 (4)	0.29
b1Bs	unif	0.15	0.4	0.28	0.28	0.27	436 (2)	0.69
b2Bs	unif	2.2	3	2.6	2.69	2.57	436 (2)	0.96
q	unif	0.5	1.5	1	1			
b1Rd^e^	unif	0.1	2	1.05	1.11^f^	0.25	32 (1)	0.37
b2Rd	unif	0.05	0.6	0.325	0.42	0.25	32 (1)	1.31
b1Ht	unif	0.05	0.15	0.1	0.11			
b2Ht	norm	0.87	0.087	0.87	0.87			
Reproduction carbon	unif	0.7	0.95	0.83	0.9			
Root beta	unif	0.0001	0.1	0.05	0.001			
SRA	unif	24	72	48	48			
*K*_max_	lnorm	−3	0.75	0.05	0.014^f^	0.12	64 (13)	0.16
*K*_exp_	norm	2	0.5	2	1.93	2.06	47 (10)	0.24
P50^e^	norm	150	50	150	206.2^f^	122.9	61 (12)	0.20
Wood capacitance	lnorm	2	0.5	0.0074	0.0017^f^	0.0083	6 (1)	0.49
Leaf capacitance	lnorm	−0.29	0.76	0.00075	0.0033^f^	0.0019	7 (1)	0.16
stoma_psi_b^e^	norm	160	40	160	192.86			
stoma_psi_c	unif	1	5	3	3			
leaf TLP^e^	lnorm	5.42	0.53	225.88	192.86	205.02	7 (1)	0.22
*V*_m0_	Weibull	1.35	40	21.47	18.75^f^	35.54	39 (2)	0.08
Quantum efficiency	gamma	4.46	59.7	0.069	0.08	0.057	19 (4)	0.49
Stomatal slope	lnorm	2.2	0.38	9.025	9	10.48	14 (1)	0.20
Root respiration	unif	0.14	0.42	0.28	0.28			
Dark respiration	gamma	2	132	0.013	0.014^f^	0.028	26 (3)	0.16
Growth respiration	beta	4.06	7.2	0.35	0.33			
mort_2_	gamma	1.2	0.058	15.36	15			
mort_3_	unif	0	0.1	0.05	0.063	0.051	18 (1)	0.50
Leaf turnover	unif	1.3	2.4	1.85	1.27			
Root turnover	Weibull	1.6	1.6	1.27	1.27			
SLA	Weibull	2.1	12.1	20.326	17.88^f^	22.06	70 (11)	0.10
rho	unif	0.1	1	0.55	0.46	0.46	66 (12)	0.07

^a^ unif = uniform distribution, lnorm = log‐normal distribution.

^b^ Only indicated when different from the median prior (i.e. when liana observations are available: *N* ≥ 0).

^c^ Number of observations (number of studies/datasets).

^d^ For the meta‐analysis of Paracou, French Guiana.

^e^ These parameters are actually negative and were multiplied by (−1) after sampling.

^f^ Indicates when the 95% CI interval of the posterior did not include the ED2 default parameter.

Parameter input uncertainty was characterized by its coefficient of variation (CV*_p_*), defined as the parameter posterior standard deviation divided by its median, $\bar{\beta_{0_{p}}}$ (Equation 1). Here and after, the overline and Var symbols indicate the median and variance operators respectively.

1. Meta‐analysis, coefficient of variation

(1) $${\text{CV}}_{p}=\frac{\sqrt{\text{Var}\left(\beta_{0_{p}}\right)}}{\bar{\beta_{0_{p}}}}$$

- Sensitivity analysis, elasticity

(2) $$\varepsilon_{p}=\left({\left(\frac{dg_{p}}{d\beta_{0_{p}}}\right|}_{\bar{\beta_{0_{p}}}}\right)\cdot \frac{\bar{\beta_{0_{p}}}}{g_{p}\left(\beta_{0_{p}}\right)}$$

- Uncertainty analysis, total parametric variance

(3) $$\text{tot}\text{.var}\;=\;\sum_{p=1}^{N}\text{Var}\left[g_{p}\left(\beta_{0_{p}}\right)\right]$$

Partial variance

(4) $$\text{rel}{\text{.var}}_{p}\;=\;\frac{\text{Var}\left[g_{p}\left(\beta_{0_{p}}\right)\right]}{\text{tot}\text{.var}}$$

Because of the limited numbers of liana allometry studies (that would be insufficient for the Bayesian meta‐analytic model to reduce model uncertainty), we used the available allometric data to generate informed priors for liana allometric parameters (slopes and intercepts).

#### 2.2.2 Sensitivity analysis

Parameters were varied one‐at‐the‐time around their median values (±1, 2, 3 *SD*) and several model responses (GPP, NPP and evapotranspiration, as well as the liana contribution to these fluxes) were fitted using a Hermite cubic spline function *g_p_*, which allowed us to estimate the model sensitivity to each parameter. Model sensitivity was estimated as the slope of the spline function $\left(\frac{dg_{p}}{d\beta_{0_{p}}}\right)$ estimated at the median parameter value, and we further computed model elasticity, defined as model sensitivity normalized by the ratio of median output to the median parameter value (Equation 2). When estimating the sensitivity of model responses, we summed up the contributions of all plants, not controlling for plant size.

#### 2.2.3 Uncertainty analysis

The outputs of the two previous steps, the parameter posterior distribution $\beta_{0_{p}}$ and the model response function *g_p_* were further used to estimate the contribution of each parameter to the model parametric uncertainty. The total parametric uncertainty was calculated as the model output variances generated by each single parameter summed up over the total number of parameters *N*, as shown in Equation 3. In this study, total and parametric uncertainty are synonymous as we only account for the latter type of uncertainty. Parameter contribution to the total parametric uncertainty *rel*.*var_p_* was then computed as the fraction of variance explained by each parameter (Equation 4).

**FIGURE 2:**
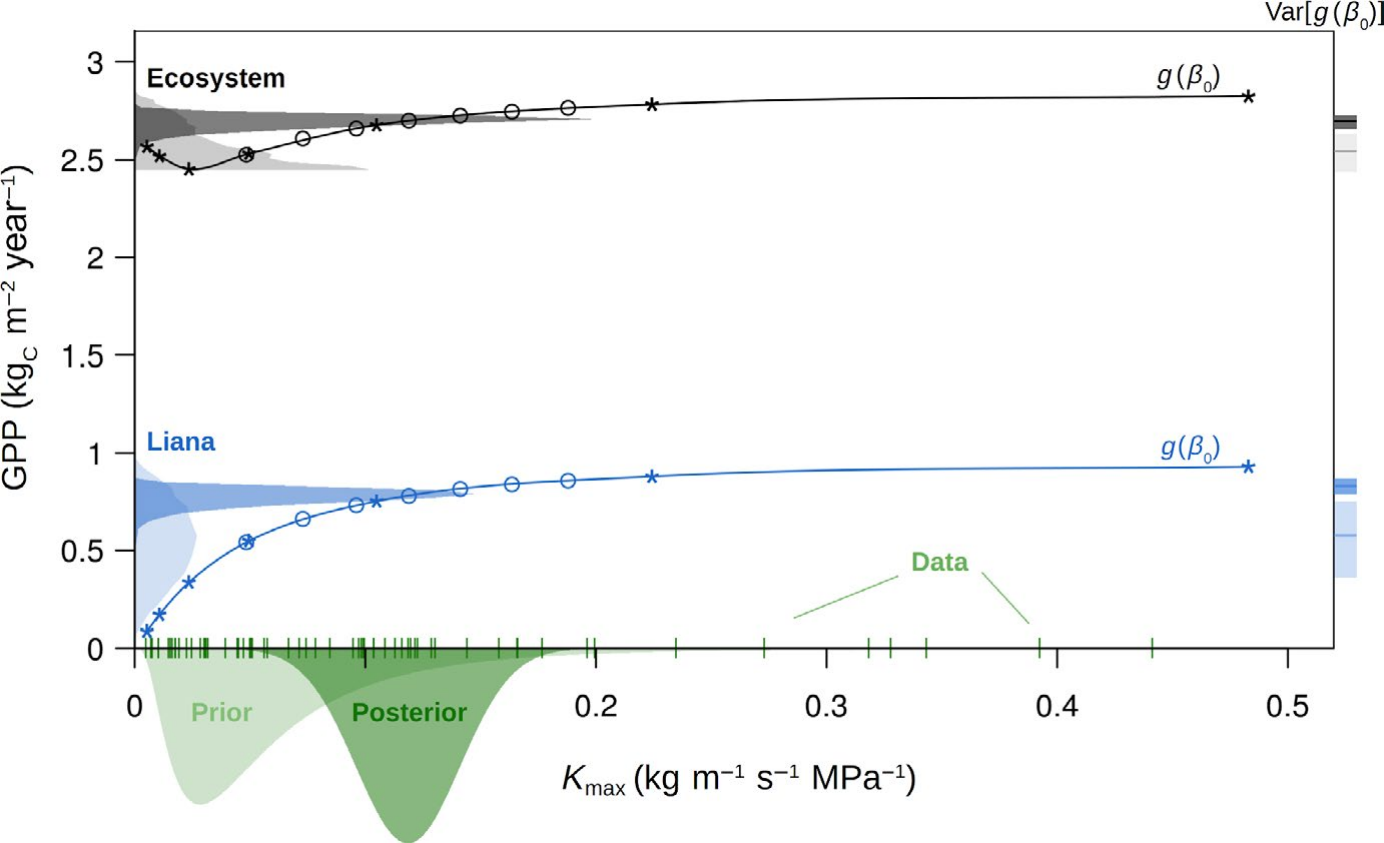
Uncertainty analysis of both the modelled ecosystem GPP (black) and its liana contribution (blue) to the liana stem conductivity *K*_max_ on BCI. The parameter prior distribution (light green) is informed by the data (vertical bars on the *x*‐axis) to generate the posterior distribution (dark green) from which different quantiles are sampled to run the sensitivity analysis and estimate the model response (blue and black dots are the ED2 model projections of the liana and the ecosystem GPP respectively). The parametric uncertainty in the model outputs is derived by transforming the posterior distributions through the spline functions g (solid lines) and is represented on the *y*‐axes by both the probability distributions (left) and their median ± 1 *SD* (right), that is, calculated as the square root of the variance Var[*g*(*β*_0_)]. For sake of completeness, the model projections are here also represented for the prior distribution (star markers), leading to larger model uncertainties and different simulated medians. All the distributions are for illustrative purposes only (they do not integrate to unity)

The entire workflow is illustrated in Figure 2 for a specific input parameter (liana stem conductivity *K*_max_) and two model responses (ecosystem GPP and its liana contribution). The hierarchical Bayesian meta‐analytic model shifts the parameter median and reduces the uncertainty by ingesting observational trait data. Model univariate sensitivity analyses (star and circle symbols) are then fitted with the spline functions to estimate the predictive uncertainties corresponding to the parameter prior (light) and posterior (dark colours) distributions. Figure 2 further illustrates how ecosystem‐scale variables can be more constrained than their individual components due to PFT compensation effects.

The benefit of the liana parameter constraining and meta‐analysis was assessed by the ratio of posterior to prior ensemble run spreads. Model output spreads were generated from ensemble simulation runs (*n* = 250), using either the prior or the posterior distributions sampled using Monte Carlo techniques (Raczka et al., 2018).

### 2.3 Site description and model setup

#### 2.3.1 Simulated sites

The model uncertainty analysis was performed for two sites: Barro Colorado Island, Panama and Paracou, French Guiana. These two specific sites were selected based on the local abundance of liana and ecosystem empirical data, their difference in liana contribution to forest biomass and rainfall regimes (Table 3; Supporting Information Figure E1).

**TABLE 3:** Main features of the two simulated forest sites

Site name	Paracou	BCI
Country	French Guiana (France)	Panama
Forest type	Tropical moist	Tropical, seasonally moist
Forest successional stage	Old growth	Old growth
Coordinates (Latitude, Longitude)	(5.3N, 52.9W)	(9.2N, 79.8W)
Mean altitude (m a.s.l.)	40	120
Mean annual temperature (°C) and interannual variability (±1 *SD*)	26.0 ± 0.3	25.6 ± 0.4
Mean annual precipitation (mm) and interannual variability (±1 *SD*)	3,088 ± 117	2,640 ± 94
Dry season	September–October (*p* < 60 mm) August/November (*p* < 100 mm)	January–March (*p* < 60 mm) April (*p* < 100 mm)
Available years of meteorological data	2004–2016	2003–2016
Liana stem density (DBH ≥ 1 cm)^a^ (ha^−1^)	126.3	1,428.9
Tree stem density (DBH ≥ 10 cm) (ha^−1^)	319.4	416.0
Liana basal area (DBH ≥ 1 cm) (m^2^/ha)	0.34	1.01
Tree basal area (DBH ≥ 10 cm) (m^2^/ha)	19.2	26.7

^a^ Liana cut‐off in Paracou inventories is 2 cm.

The forest of BCI is an old‐growth seasonally moist lowland tropical forest with an average annual rainfall of about 2,640 mm (Detto et al., 2018) and a well‐marked dry season (total rainfall between late‐December and mid‐April is about 175 mm on average). Located on the coastal part of French Guiana, the Paracou research station is classified as a lowland moist primary forest (Aguilos et al., 2018; Bonal et al., 2008; Malhi, 2012) which, compared to BCI, experiences higher precipitation rates (recorded mean annual precipitation is almost 3,100 mm), and a weaker and shorter dry season spanning from mid‐August to mid‐November (total rainfall during this period is 238 mm). Both sites support tropical evergreen moist forests and we therefore imposed an evergreen phenology to all plant functional types of this study, following Powell et al. (2018) and di Porcia e Brugnera et al. (2019).

#### 2.3.2 Prescription of atmospheric forcings

For both sites, we used the meteorological data from the local flux tower measurements as atmospheric forcings (see Table 3 for respective spanning periods) and used the observed carbon and energy exchange fluxes obtained with the eddy‐covariance method to benchmark the modelled productivity and evapotranspiration (Aguilos et al., 2018; Bonal et al., 2008; Powell, Kueppers, et al., 2017). Meteorological data of the simulated years were readily available at hourly resolution for air temperature, wind speed, specific humidity, precipitation rate, short‐ and long‐wave radiation and were hence used as ecosystem upper boundary condition. To exclude CO_2_ fertilization effects and keep the same meteorological drivers as in our previous study (di Porcia e Brugnera et al., 2019), the atmospheric concentration of CO_2_ was fixed at a constant value of 370 ppm, which corresponds to initial concentrations measured by the flux towers.

#### 2.3.3 Vegetation initial conditions

Model simulations were initialized with local liana and tree inventories. On BCI, we used the 50‐ha inventories of lianas and trees of 2007 and 2010 respectively. They include all trees and lianas with DBH ≥ 1 cm in the 500 m × 1,000 m plot (Condit et al., 2019). The 50‐ha site was divided in a regular grid of 20 m × 20 m, which resulted in an initial number of 1,250 patches that were allowed to fuse during the first model time step. In Paracou, tree and liana censuses come from 10 inventory plots of 70 m × 70 m established in the flux tower footprint in 2004 that include all large individuals (for trees: DBH ≥ 10 cm; for lianas: DBH ≥ 2 cm since 2015).

At both sites, trees were classified in one of the three tropical tree PFTs according to their wood density, as estimated by merging the tree species lists with the Global Wood Density Database (Zanne et al., 2009) and using ED2 mid‐range values as class separators.

Because censuses were not available for trees <10 cm DBH at Paracou, we extrapolated the number of tree individuals in the 1–10 cm DBH class range using a linear model applied to the log–log transforms of the DBH size class versus the plant density. We filled the missing class of trees by generating the estimated number of plants from the three tropical tree PFTs based on their relative frequency in the inventory.

From liana inventories, it appeared that liana density was much higher on BCI and not only because the inventory in Paracou did not include smaller lianas comprised between 1 and 2 cm (Table 3). Paracou counted a few more large (DBH > 14 cm) lianas (four individuals ha^−1^) as compared to BCI (two individuals ha^−1^). However, there were considerably less small (2 ≤ DBH ≤ 14 cm) lianas in Paracou (123 individuals ha^−1^) than on BCI (669 individuals ha^−1^).

On BCI, large liana (DBH > 3 cm) mean density (320 individuals ha^−1^) was comparable to large tree (DBH > 10 cm) mean density (410 individuals ha^−1^). Yet, liana density largely varied from liana‐free (<5 ha^−1^) to liana‐infested patches (up to 1,100 lianas ha^−1^).

Similarly, areas with different levels of liana infestation co‐existed in Paracou: Large liana density ranged from 0 to 210 individuals ha^−1^. On the landscape average, large lianas (80 individuals ha^−1^) were less abundant than large trees (324 individuals ha^−1^).

Here and everywhere in the manuscript, we refer to liana stems (ramets) as liana individuals while they are not always individuals in the genetic sense (i.e. genets).

#### 2.3.4 Competition and model scenarios

To determine the driving force of competition between lianas and trees, we classified each liana parameter according to its relevance for below‐ground (water) or above‐ground (light) competition (Table 1). We also classified them by plant organ (leaf, stem, root, seed or entire plant for parameters that could not be primarily related to a single organ) and ecophysiological process (allocation, water use, photosynthesis, respiration, mortality, tissue turnover or structural parameters), see Table 1. Tissue turnover represents the maintenance costs of leaves and roots. Summing up the relative contribution of all parameters belonging to each group (of process, organ, competition types) allowed us to determine the most critical parameter categories for uncertainty.

All model simulations were run for 5 years. ED2 was run as standard and all patches and cohorts were allowed to age, grow or disappear. To investigate competition shifts over time, we assessed the model uncertainty both over the full simulation duration and during dry periods only. We defined as dry the months during which rainfall did not exceed 100 mm. To account for competition changes across forest stand ages, the uncertainty analyses were run starting either from the full set of initial conditions or from young forest patches only. Because forest inventories did not provide any information on age, we assumed patch age based on three criteria: the initial liana density, the initial abundance of late successional trees and the initial patch height. Thresholds of these criteria were progressively modified from the most extreme values to include a minimum of five patches on BCI (of 1,250) and one (of 10) in Paracou. By doing so, we ended up selecting six patches on BCI and one in Paracou in which the liana initial density was among the highest in the respective sites, alongside with a disproportionately low initial representation of late successional trees in patches that were initially shorter than the average. Distributions of these criteria are represented for both sites and the selection of young patches from the inventory highlighted in the 50‐ha plot of BCI in Supporting Information Figure E2.

The uncertainty analysis and the model runs were all achieved using PEcAn (pecanproject.org, workflow IDs 99000000674 to 99000000680). We also simulated the same sites under the same conditions but without lianas in order to evaluate simulated changes in forest dynamics without the liana‐tree competition. The no‐liana simulations were simply run by removing liana cohorts from vegetation initial conditions and turning off the liana PFT.

## 3 RESULTS

### 3.1 Parameter distributions and meta‐analysis

**FIGURE 3:**
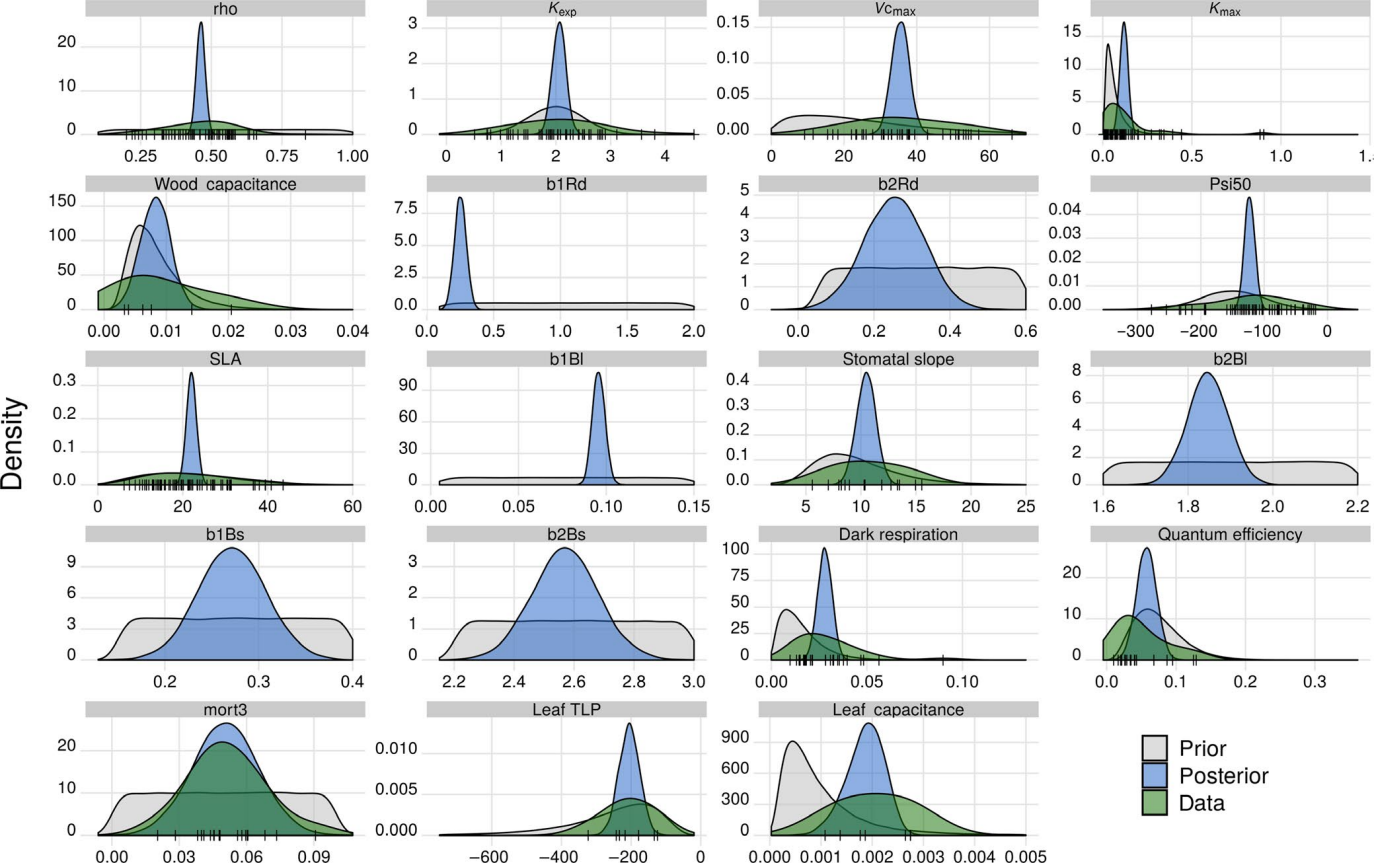
Liana PFT parameter distributions. The prior distributions (grey) are relatively broad and were established to encompass natural variability of parameter values and cover all field observations (black vertical lines smoothed into the green distributions). A Bayesian meta‐analysis was performed to combine the prior distributions with trait data (whenever available) to create posterior distributions (blue), which were further used to estimate both the model and parameter uncertainties. The figure only illustrates the parameter distributions for which data were available. Note that we did not use the Bayesian meta‐analysis for the allometric parameters (b1Rd, b2Rd, B1Bl, B2Bl, b1Bs and b2Bs). Instead, a posterior distribution constrained to data was directly built for each of those. The units of each parameter are given in Table 1

We were able to collect data for 19 of the 32 liana parameters we selected in this study (Figure 3; Table 2): six hydraulic, six allometric, four photosynthetic and two structural parameters as well as the density‐independent (i.e. ageing) mortality rates of lianas that were extracted from Phillips et al. (2005). The priors of the remaining 13 parameters could not be constrained by data. In Table 2, parameters with a posterior median were constrained by observations through the Bayesian meta‐analysis. The number of traits that were ingested by PEcAn meta‐analysis varied from very low numbers in one single study (wood and leaf capacitances, turgor loss point) to large number in a wide collection of papers (such as the stem hydraulic conductivity with 64 sample means from 13 different scientific studies, or the specific leaf area with 70 trait data means from 11 studies), see Table 2. Liana allometric observations from four different studies served to constrain six priors related to rooting depth (*b1Rd* and *b2Rd*), structural woody biomass (b1Bs and b2Bs) and leaf biomass (*b1Bl* and *b2Bl*) allometries (Table 2; Supporting Information Figures F1 and F2).

In many cases, liana model default parameters (i.e. the ones used in di Porcia e Brugnera et al., 2019) were substantially altered by the literature meta‐analysis (Table 2). Posterior median photosynthetic capacity *V*_mc0_ was twice its original value (when lianas were parameterized as early‐successional tropical trees), just as the dark respiration factor posterior median was twice its default value (for which lianas were considered as all C3 plants). Posterior median stem conductivity was increased by one order of magnitude, while liana vulnerability to cavitation increased as compared to the ED2 default calculation derived from tropical trees (Christoffersen et al., 2016).

Liana rooting depths estimated from posterior distributions were considerably shallower than the ones that used default allometric coefficients (Figure 3; Table 2): Liana default rooting allometric coefficients were assumed to be similar to tropical trees and hence not based on observational data. As both the allometric intercept and slope of the height‐rooting depth relationship were reduced driven by destructive observations in a dry forest in Costa Rica (Smith‐Martin et al., 2019), the meta‐analysis confined the liana root biomass to the first metre of soil instead of the default deep‐rooted lianas (Supporting Information Figure F2). As lianas often reached the top canopy, they were among the tallest plants, and hence the deepest rooted plants in ED2 default simulations, consistent with traditional assumptions in the literature (de Azevedo Amorim et al., 2018; Schnitzer, 2005) while recent experimental findings revealed shallower liana root systems (De Deurwaerder et al., 2018; Smith‐Martin et al., 2019).

With a combination of higher specific leaf area, higher leaf biomass allometric intercept coefficient and a lower leaf biomass slope coefficient, the meta‐analysis posterior parameter medians predicted a lower leaf area for large liana individuals (DBH > 8.3 cm) and a larger leaf area for small lianas (DBH < 8.3 cm) than the model default.

In Paracou, the ratio of the parameter coefficients of variation after and before meta‐analysis was always lower than 1 except for the rooting depth slope allometric coefficient (*b2Rd*). The standard deviation of *b2Rd* was also reduced after data ingestion (i.e. stronger constraints), but this effect was overcompensated by a large decrease of the distribution median (Table 2). This indicates that the posterior distributions were systematically more constrained than the a priori distributions of the model parameters. The posterior to prior CV_p_ ratio varied between 0.07 (*b1Bl*, *rho*) and 1.31 (*b2Rd*) with a mean of 0.35. These results were essentially the same for BCI.

### 3.2 Ensemble runs and liana impacts on forest

In both sites, the model could capture many of the structural characteristics of the ecosystems. On BCI, the simulated total leaf area (LAI, 4.6 ± 0.3 for the posterior ensemble runs) and the above‐ground carbon stocks (AGB, 14.4 ± 0.3 kg_c_/m^2^) were in line with the quantities observed by Schnitzer and Carson (2010) and Powell, Wheeler, et al. (2017): 4.8 ± 0.5 and 14.0 ± 0.1 kg_c_/m^2^ respectively. Here and everywhere in the manuscript, the error terms represent one standard error. The simulated LAI values were lower than plant area index observations achieved in the closeby site of Gigante (Rodríguez‐Ronderos et al., 2016, mean of around 6 m^2^/m^2^) but those also included wood area index. In Paracou, the simulated LAI was in agreement with observed values (4.1 ± 0.3 vs. 4.9 ± 0.9) from Cournac et al. (2002), but the simulated above‐ground biomass from the posterior ensemble runs was lower than observed (12.8 ± 0.2 vs. 17.3 ± 3.1 kg_c_/m^2^, see Ho Tong Minh et al. (2016). The latter resulted from the use of ED2‐default tree allometric coefficients rather than site‐specific ones.

On average, simulated lianas accounted for about one‐fourth (24%) and one‐eighth (12.5%) of the landscape average leaf area on BCI and in Paracou, respectively, while accounting for less than 3% of the above‐ground biomass in both ecosystems (2.8% and 1.6%, respectively). Those numbers are averages across ensemble runs and over the duration of simulation. However, liana abundance did not dramatically change over time and hence neither did liana contribution to forest biogeochemical cycles (Supporting Information Figure F5). After 5 years, liana density on BCI remained higher than in Paracou (0.14 liana m^−2^ vs. 0.013 liana m^−2^), which is in agreement with observations/initial conditions (Table 3).

**FIGURE 4:**
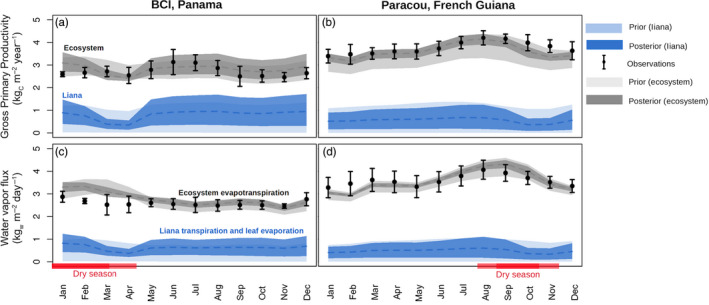
Seasonal fluxes of gross primary productivity (subplots a and b) and water vapour flux (subplots c and d) in both BCI, Panama (subplots a and c) and Paracou, French Guiana (subplots c and d) as observed by flux tower measurements (black dots) or as simulated by ED2 (envelopes). The error bars for the observations indicate the inter‐annual variability (mean ± 1 *SD*) while the envelopes represent the spread in the simulation at the ecosystem level (grey) or for the liana PFT (blue) when using either the prior (light) or the posterior (dark) parameter distribution. The liana water vapour flux is the sum of the liana PFT transpiration and leaf evaporation while the ecosystem water vapour flux is the sum of all PFTs transpiration, leaf evaporation and soil evaporation. The red envelopes highlight the local dry season (light = mean monthly precipitation <100 mm, dark = mean monthly precipitation <60 mm). Observations were averaged over 2003–2016 (BCI) and 2004–2016 (Paracou) while simulations were averaged over the 5 years of runs (2004–2009)

The model reproduced gross ecosystem fluxes both on BCI and in Paracou, as well as their seasonality, with a small overestimation of the water vapour flux during the dry season on BCI (Figure 4, the evapotranspiration RMSE is 0.58 kg_w_ m^−2^ day^−1^ for the January–April dry period during which mean observed water flux is 2.65 kg_w_ m^−2^ day^−1^). Median runs from the posterior distributions led to relatively small yearly RMSE of observed versus simulated ecosystem GPP (0.25 and 0.22 kg_C_ m^−2^ year^−1^ on BCI and in Paracou, which corresponds to 9.2% and 8.7%, respectively, of the mean observed gross primary productivity) and latent heat (0.32 and 0.26 kg_w_ m^−2^ day^−1^, which corresponds to 8.6% and 7.3%, respectively, of the mean observed evapotranspiration). Posterior flux estimates were all improved as compared to prior median runs, with the exception of the ecosystem GPP on BCI (yearly RMSE of 0.21 vs. 0.25 kg_C_ m^−2^ year^−1^ for the prior and the posterior distribution medians). The estimate of the seasonal cycle of evapotranspiration on BCI was substantially improved when using posterior ensemble runs (yearly RMSE of 0.32 vs. 0.41 kg_w_ m^−2^ day^−1^ for posterior and prior ensemble median respectively).

According to the model simulations, lianas were responsible for an important part of the ecosystem GPP (on average 28% and 15% on BCI and in Paracou, respectively, for the posterior runs) and evapotranspiration (23% and 13%), see Figure 4. The model predicted an important reduction of the liana carbon and energy exchange fluxes in both sites towards the end of the dry season (liana GPP reached 58% of its yearly mean value in April on BCI, 67% in November in Paracou) as a consequence of their shallow root system and the negative water potential their leaves experienced when the upper soil layers dry out (Figure 4). This reduction of photosynthetic activity due to water uptake limitation was less strong in the posterior runs in which lianas were characterized by higher stem hydraulic conductivity and larger photosynthetic capacity that prevented them to die‐off. On the contrary, some of the prior runs predicted a complete liana extinction because of a too large reduction in carbon gains: The minimum contribution of lianas to GPP and evapotranspiration then reached zero (Figure 4).

All confidence intervals (CI) of the ensemble runs were reduced after meta‐analysis, especially for the landscape average variables. This indicates a successful parametric constraining through the meta‐analysis. Ecosystem and liana LAI CI spread decreased by more than 45% in both sites over the entire duration of the simulation (Supporting Information Figure F5) and ecosystem AGB CI decreased about 75%. In addition, the reduction in ecosystem flux CI was on average about 70% in Paracou and between 30% and 50% on BCI (GPP and latent heat respectively). The reduction in liana flux uncertainty was around 20% yearly in both sites, and reached 60% on BCI and 40% in Paracou during the dry season (Figure 4).

Lianas negatively impacted tree growth, mortality and forest productivity by increasing water and light competition in our simulations. When including the liana PFT in simulation runs, tree growth was reduced on average by 40% on BCI and 30% in Paracou, driven by a reduction of the total tree GPP (about 25% in both sites). The reduction in tree productivity was not compensated by the additional liana carbon uptake. The overall net ecosystem productivity was dramatically reduced in both sites with the liana PFT activated: BCI switched from a neutral ecosystem to a net carbon source (NEP decreased from −0.01 to −0.30 kg_C_ m^−2^ year^−1^) and Paracou carbon sink strength declined (NEP decreased from 0.75 to 0.48 kg_C_ m^−2^ year^−1^). It was mainly the early‐successional trees that suffered from the competition (their GPP was reduced on average by 40% in both sites). Trees experienced higher drought stress levels and hence larger mortality rates due to negative carbon balance on BCI than in Paracou. Tree mortality decreased in both sites when lianas were removed (by 30% and 0.5%, on BCI and in Paracou respectively). Those results are in line with increased tree sapflow velocity (Alvarez‐Cansino et al., 2015), wetter shallow soil layers (Reid et al., 2015) and less negative tree leaf water potentials (Pérez‐Salicrup & Barker, 2000), observed right after liana removal.

### 3.3 Uncertainty analysis and competition factors

In this section, we explore the outputs of the uncertainty analysis of the liana PFT. We mainly illustrate these results using the liana contribution to ecosystem GPP as model output since this represents the capacity of lianas to maintain, grow or thrive through competition with the tree PFTs. As detailed below, results for other fluxes such as evapotranspiration and NPP are very similar.

**FIGURE 5:**
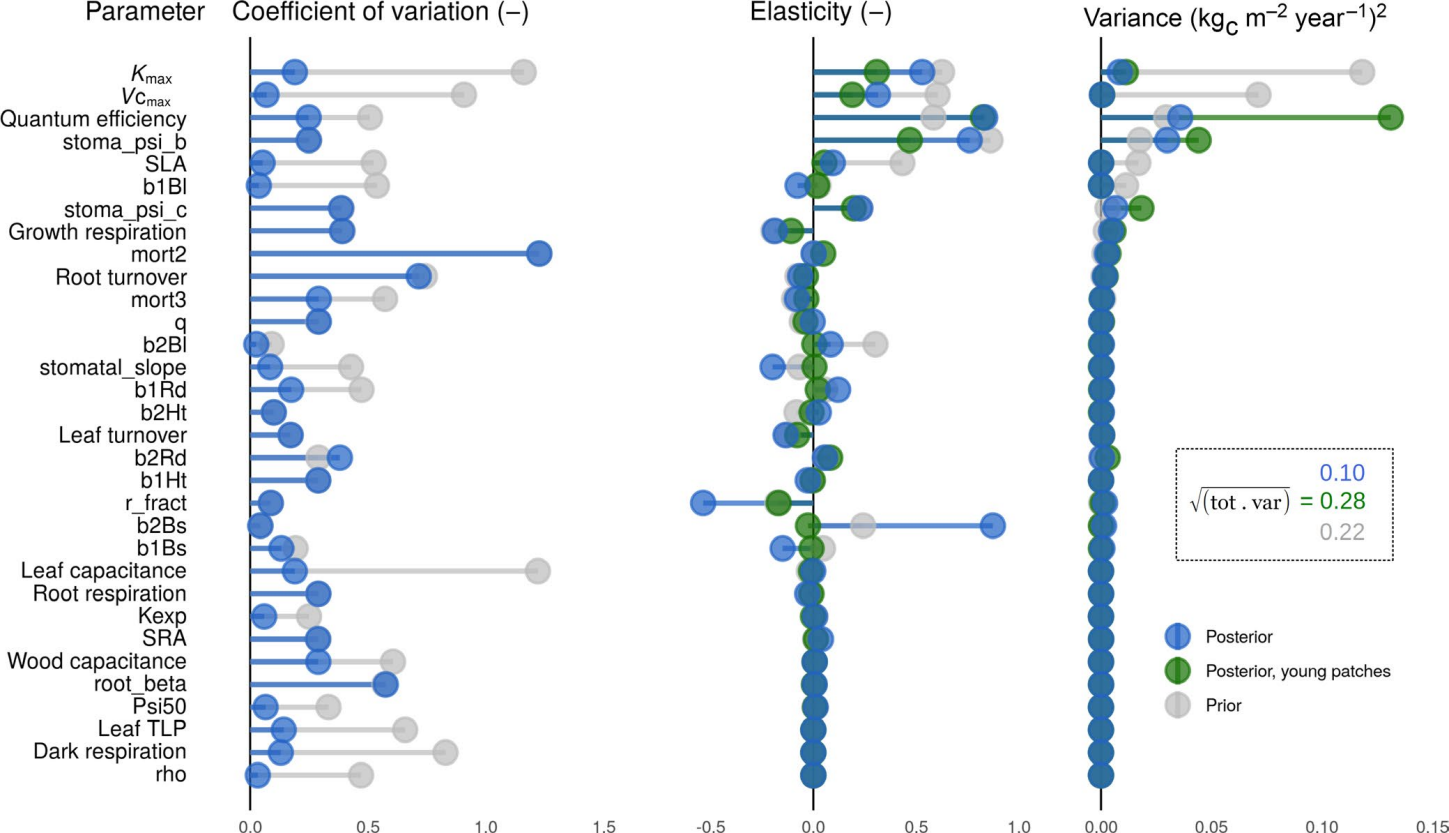
The liana parameter contribution to the 5‐year time‐scale model uncertainty of the liana GPP, decomposed as the parameter uncertainty (coefficient of variation, left panel), model sensitivity (elasticity, middle panel) and model uncertainty (variances, right panel) for the prior (grey), posterior (blue) and posterior in young patches only (green) on BCI, Panama. Parameter description, units and prior/posterior distributions can be found in Tables 1 and 2 and Figure 3. The total standard deviation of the liana GPP was reduced from 0.22 (prior) to 0.10 (posterior) kg_C_ m^−2^ year^−1^. The parameters were sorted by their partial variance contribution for the prior distributions

After integration of the available data during the meta‐analysis, liana photosynthetic quantum efficiency (with a relative contribution of 37%) and the stomatal closure regulation parameter *stoma_psi_b* (31%) were the strongest drivers of liana GPP uncertainty on BCI, with all other parameters contributing <10% to the overall uncertainty (Figure 5). Not only did the relative contribution of these two parameters increase after meta‐analysis, but so did the model output absolute variances generated by them, which is explained by a larger model sensitivity (steeper slope) of the model around the posterior median parameter set (Figure 5).

**FIGURE 6:**
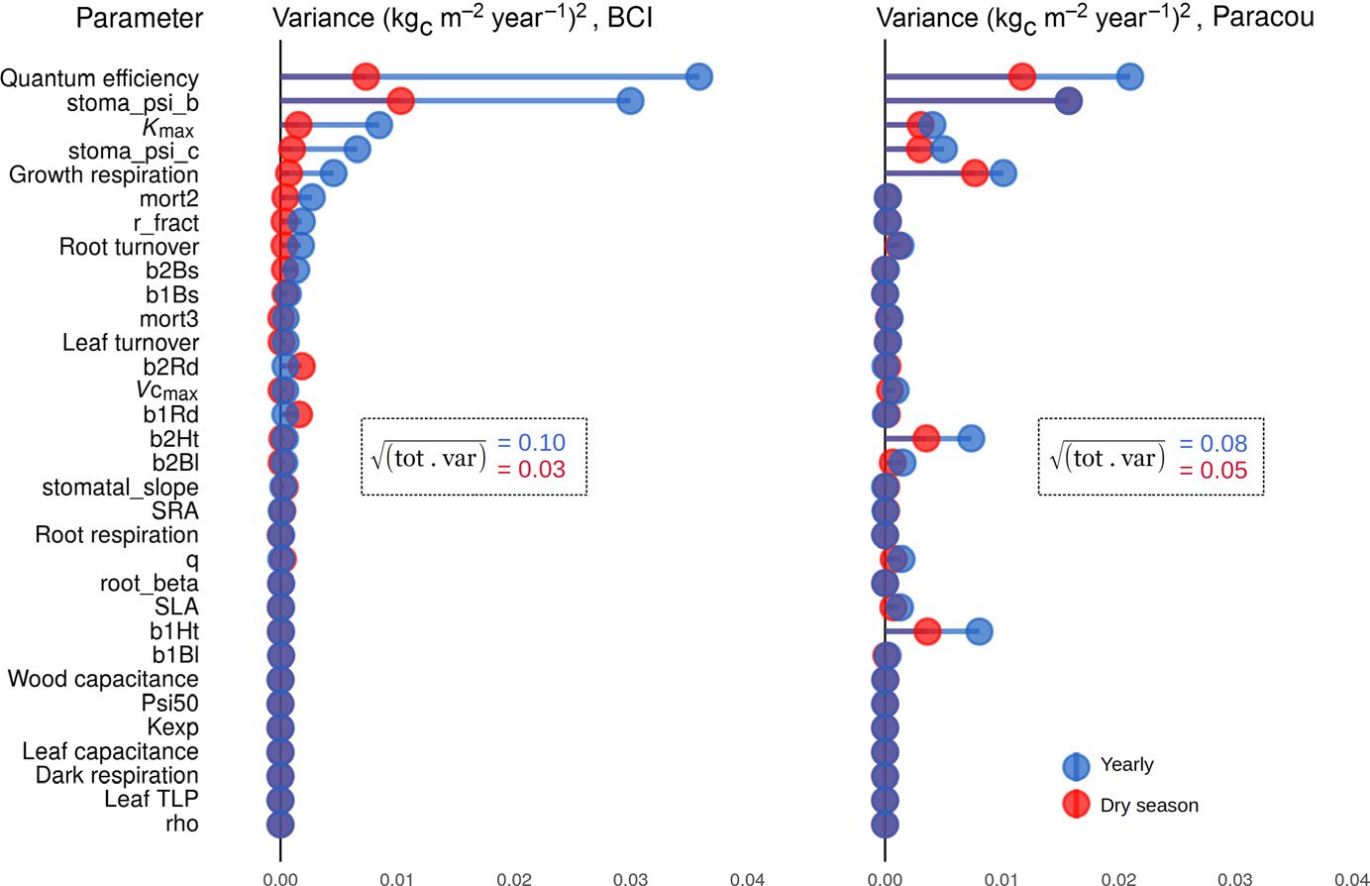
Comparison of the contribution of the liana parameters to model uncertainty (here, the liana GPP) during the dry season (red) or yearly (blue) on BCI, Panama (left) and Paracou, French Guiana (right). The parameters are sorted by their contribution to uncertainty on BCI over the entire year, which is the posterior shown in the last panel of Figure 4. The total standard deviation (the square root of the sum of the variances) is also given for each single scenario

Our results indicate that the contribution of liana parameters to explain the variability of liana GPP was season dependent. While liana quantum efficiency and stomatal regulation parameter (*stoma_psi_b*) remained the most critical parameters for liana GPP throughout the year, their contribution to the model output variance systematically decreased during the dry season (Figure 6). Liana quantum efficiency and *stoma_psi_b* set aside, height allometry coefficients (*b1Ht* and *b2Ht*) and growth respiration appeared to be the most critical factors at any time in the wetter site of Paracou (Figure 6). Root biomass allocation parameters (*b1Rd* and *b2Rd*) appeared more critical in the model uncertainty analysis during the dry season of the more water‐limited site (BCI), while water transport (*K*_max_) and stomatal regulation (*stoma_psi_c*) were almost equally important during all seasons in Paracou (Figure 6).

**FIGURE 7:**
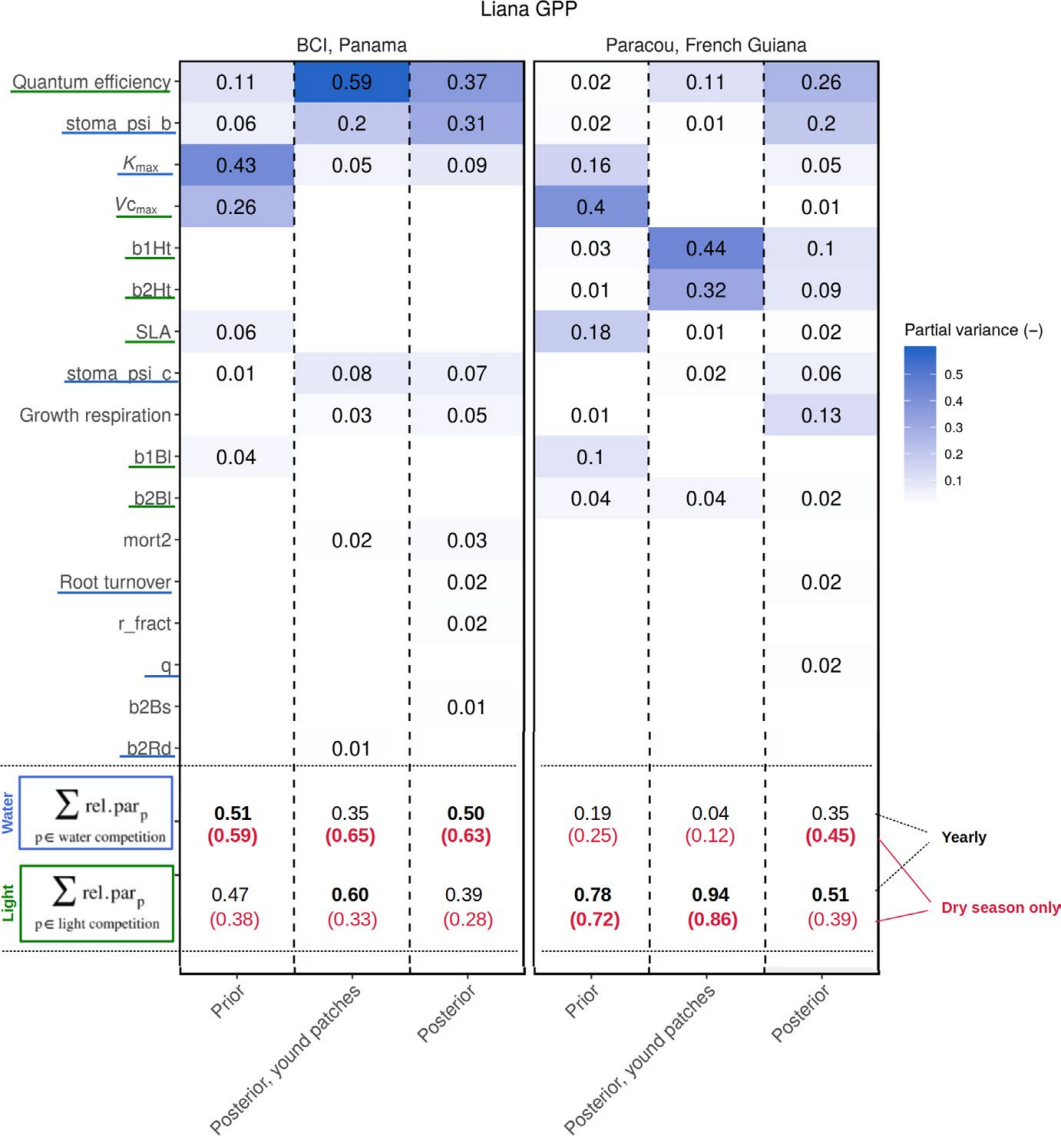
Relative contribution of liana parameters to liana GPP model uncertainty (relative variances, rel.par_p_) in both BCI, Panama (left) and Paracou, French Guiana (right) for the prior, posterior and posterior in young patches only distributions. Parameters were ordered by their partial variance contribution summed up over both sites and all three scenarios. Only the contributions superior to 1% (in at least one of the sites or one of the scenarios) are shown in the figure. In addition, the left column shows the classification into water‐related (blue underlines) and light‐related (green underlines) parameters as determined in Table 1. Finally, parameter relative contributions to both competition types (water and light) are presented considering entire simulations (black, which is basically the sum of the relative contributions presented just above) and during the dry season only (red, single relative parameter contributions not shown). The competition type dominating for each particular scenario and site is presented in bold

In younger patches, the overall output uncertainty was much larger than over the whole forest ecosystem (Figure 5, 0.28 vs. 0.10 kg_C_ m^−2^ year^−1^). Notwithstanding, the ranking of parameter contribution to overall variance (Figure 5, right panel) remained similar to the landscape average: *quantum efficiency* (59% of the total parametric variance) and *stoma_psi_c* (20%) still led the uncertainty and were followed by *stoma_psi_b* (8%) and *K*_max_ (5%). In these younger parts of the forest, abundant lianas are strongly competing with fast‐acquisitive early‐ and mid‐successional tree PFTs, which are more abundant in young patches than in the overall ecosystem. Consequently, slight changes of liana quantum efficiency, hydraulic conductivity (*K*_max_) or stomatal regulation (*stoma_psi_b* and *stoma_psi_c*) generate big variations of liana to tree competition: Lianas can either suddenly arrest the succession or rapidly disappear. Liana *quantum efficiency* and stomatal regulation (*stoma_psi_b*) systematically led the model uncertainty of liana GPP, except for young patches in Paracou (Figure 7) where liana height allometric coefficients (*b1Ht* and *b2Ht*) drove the plant competition for light resources.

After aggregating parameters into water versus light competition, water competition appeared to be the most important factor of liana GPP (50%) on BCI while light competition dominated the uncertainty in Paracou (51%, Figure 7). The dry season reinforced the impact of water‐related traits and systematically increased the contribution of this category of parameters to the total parametric variance (63% and 45% on BCI and in Paracou for the posterior runs, Figure 7). Even in the wetter site (Paracou), water dominated as the most critical resource during the dry season (even if in that case, both contributions to competition were close: 45% and 39%, for water and light respectively). Finally in young patches, light was systematically the most critical resource in Paracou (94% for yearly averages, 86% for the dry season only, Figure 7) while it fluctuated between water (dry season, 65%) and light (wet season, 60%) on BCI.

The trends detailed above (increasing contribution of water‐related parameters during the dry season, switch of the dominating competition factor in Paracou over seasons, critical importance of the water acquisition on BCI, discrepancies between younger and older forest patches) were also valid for other important output variables. The contribution of the parameters to the liana evapotranspiration correlated very well with their contributions to the liana GPP, once aggregated into the competition factors (*r* ^2^ = 0.91, slope = 1.001). For the liana contribution to the ecosystem NPP, the growth respiration parameter played a very important role (mean partial variance of 37% for the uncertainty analysis of posterior runs across sites and seasons, see also below). This was similarly found for other tree PFTs in ED2 and relates to the model structure (see Section 4). This parameter aside, partial variances of liana parameters for the contribution of lianas to GPP and NPP were also very well correlated (*r* ^2^ = 0.84, slope = 1.12). Therefore, the conclusions drawn above for liana GPP remain valid for modelled fluxes of liana NPP and evapotranspiration.

**FIGURE 8:**
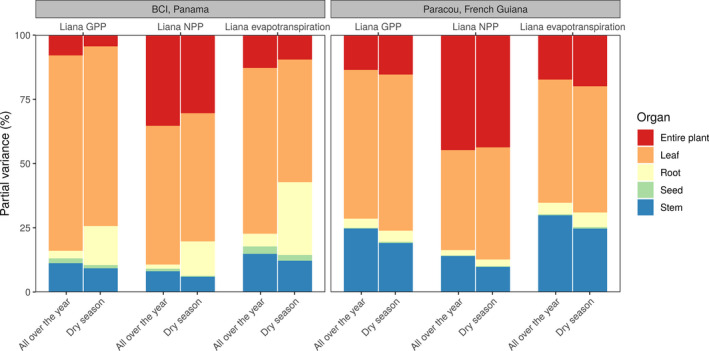
The relative contribution of liana parameters to liana evapotranspiration, GPP and NPP model uncertainty (partial variances) on BCI, Panama (left) and Paracou, French Guiana (right) over the whole year or during the dry season as aggregated by organ according to the classification of Table 1

**FIGURE 9:**
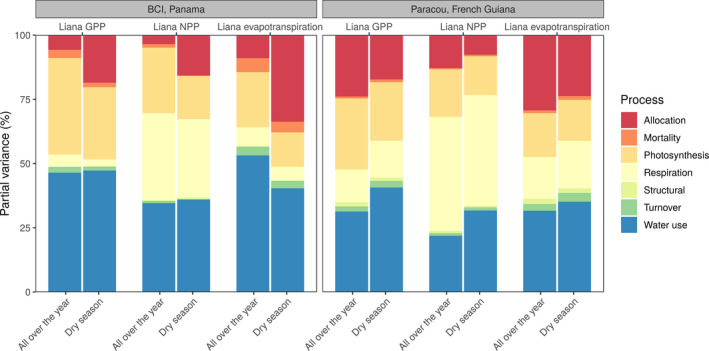
The relative contribution of liana parameters to liana evapotranspiration, GPP and NPP model uncertainty (partial variances) on BCI, Panama (left) and Paracou, French Guiana (right) over the entire year or during the dry season as aggregated by process according to the classification of Table 1. Turnover indicates the living tissue maintenance costs

While the partial variances of liana parameters varied over time and between forest sites and stand ages, the contribution of the different plant organs and processes remained relatively consistent for the different model outputs (Figures 8 and 9). On BCI, the leaf‐related parameters (60% on average Figure 8) and water use‐related parameters (43%, Figure 9) overall dominated model uncertainties, even though respiration‐related parameters (driven by the growth respiration parameter) became almost as important as water use for liana NPP (32% vs. 35%, Figure 9). During the dry season, allocation parameter contribution (driven by rooting depth allometric coefficients) increased (+17% on average) while water use‐related parameters either remained constant (liana GPP and NPP) or decreased (−13%, liana evapotranspiration), so that in total, water‐related parameter contribution always increased during the dry season.

In Paracou, leaf organ importance systematically decreased in favour of entire plant‐scale parameters (especially *b1Ht* and *b2Ht*): on average, leaf‐related parameters contributed to 49% of the total variance and plant‐scale parameters to 26%. On BCI, the contribution of these leaf‐related parameters and plant‐scale parameters reached 60% and 16%, respectively. Similarly, Paracou was characterized by a higher contribution of allocation parameters as compared to BCI (+6% on average) at the expense of water‐use parameters (−11%), driven by growth for light competition and the height allometric coefficients.

## 4 DISCUSSION

### 4.1 Liana impact and competition across simulated sites and forest stand ages

This study is an important step towards realistically representing lianas in vegetation models. Our approach completes the first attempt to include the lianescent growth form in ED2, as it fills several gaps in the previous study (di Porcia e Brugnera et al., 2019). Primarily, it mechanistically accounts for the hydraulic architecture differences between lianas and trees, as observed by many studies (Ewers et al., 1990; Johnson et al., 2013; Maréchaux et al., 2017; Tyree & Ewers, 1996; van der Sande et al., 2013, 2019; Zhu & Cao, 2009) and therefore allows us to extend the use of such a model to drier sites or to more extreme (i.e. future) climatic conditions. It also targets shorter time‐scales as compared to the original publication (years vs. centuries) to focus on the mechanistic processes driving intergrowth form competition. Moreover, we extended the use of the liana PFT to prescribed initial conditions in addition to near‐bare ground initialization. It is worth noting that the liana PFT is slightly different to the one used for the production runs in the original publication (di Porcia e Brugnera et al., 2019) as liana height limitation was here applied at the patch level rather than at the cohort level (see Appendix C for more details). Furthermore, the new version of the liana PFT was parameterized using the most up‐to‐date observational data as opposed to the default pioneer tree parameters that were used before (see Figure 3; Table 2).

The model simulations presented in this study captured many features of two tropical forests characterized by contrasting amounts and seasonality of rainfall, as well as liana abundance. Both forest structural properties (total LAI and AGB, Figure E5) and flux measurements derived from eddy‐covariance observations (GPP and latent heat, Figure 4) were well reproduced in simulation runs, which increased our confidence in the model predictions. In addition, integrating existing liana trait data (Figure 3) made the model ensemble runs converge towards observed ecosystem gross productivity and evapotranspiration, reduced the flux and pool confidence intervals (Figure 4; Figure E5) and reduced the overall model uncertainties (Figure 5).

The impact of lianas on forest dynamics was also reproduced by model simulations. By strengthening competition for below‐ground resources, lianas increased the simulated drought stress experienced by trees, especially during the dry season, as experimentally observed in liana removal experiments (Alvarez‐Cansino et al., 2015). Liana removal triggered tree drought‐stress relief in the simulations, as suggested by experimental data (Pérez‐Salicrup & Barker, 2000; Reid et al., 2015). Overall tree growth considerably increased in both sites when removing the liana PFT from the simulations (+30%–40%), which is in line with observed tree growth increases after liana removal (van der Heijden et al., 2015). Similarly, the predicted increase in tree mortality (+30% on BCI) relative to liana‐free simulations is confirmed by experimental observations (van der Heijden et al., 2015). In the model, the increase in mortality was caused by a reduction of carbon gains for trees when lianas were added to the runs. This, in turn, was due to a combination of decreased tree stomatal conductance due to drought stress (below‐ground competition) and declined light interception by tree PFTs (above‐ground competition) caused by lianas. Liana removal in the simulations led to forest recovery, enhanced forest productivity and recovered sink strength, just like in the experimental plots (van der Heijden et al., 2015). According to the model, the effect of lianas on the forest does not differ between seasons: The strengths of water and light competition compensate each other over time, as observed experimentally (van der Heijden et al., 2019). The vegetation model also enabled disentangling the contrasting impact of lianas on the forest composition: abundance and productivity decreased more in early successional trees than in the other tree PFTs because the former shared more similar ecological niches (fast acquisitive, low wood density, high mortality rates).

Water competition played a more important role than hypothesized. In silico, the competition between growth forms was dominated by water acquisition all year long on BCI and during the dry season in Paracou (Figure 7), even though the two selected sites were quite wet (Table 3). Several seminal studies investigating growth forms competition already indicated that water is critical for determining the impact of lianas on forest dynamics (Andrade et al., 2005; De Deurwaerder et al., 2018; Tobin et al., 2012). Our numerical findings reinforce the idea that below‐ground competition is crucial in liana tree relationship as water acquisition dominated the competition even during the relatively short and weak dry season in Paracou. In sites characterized by lower yearly rainfall, and hence higher liana densities (DeWalt, 2010; Schnitzer, 2005; Schnitzer & Bongers, 2011), the relative importance of below‐ground competition is expected to increase even more than we found for BCI and Paracou (Figure 7). The relative contribution of light competition that we observed (during the wet season and in young patches in both sites) decreased with decreasing water availability (Figure 7) and will probably keep doing so in drier conditions. Therefore, the simulated relative contributions of water to the liana‐tree competition (35% and 50% in Paracou and on BCI) are likely to be on the low side, and could increase if stronger seasonality or decreased precipitation is expected in the future.

Despite the fact that we only included two sites in this analysis, the modelling workflow and the new model development allow expanding simulations over a larger rainfall gradient in the future. Next steps should focus on the ability of ED2 to reproduce trends of liana abundance with dry season length and mean annual precipitation (Schnitzer & Bongers, 2011) or seasonality (Parolari et al., 2020) over a larger number of sites. This expansion to drier conditions should confirm the observed trend in this study of water dominating the liana versus tree competition.

### 4.2 Uncertainty analysis and key parameters

The Bayesian workflow that we applied here and that was developed in previous studies (LeBauer et al., 2013) allowed (a) constraining key ecophysiological parameters both directly from meta‐analysis of trait data (as we did in this study) or inversely from ecosystem‐level observations (ongoing research) and (b) identifying the most important parameters for both liana productivity and its competition with tropical trees. It did so by merging all existing observational data (regardless of the site, dataset size or the liana species) and explicitly accounting for observational and parametric uncertainty in a straightforward way. Constraining model parameters to data shifted liana default parameterization (Table 2), led to a more realistic representation of the lianas and reduced model uncertainty (Figure 4). Moreover, it should guide future field data collection: Quantifying the relative parameter uncertainties revealed the most critical inputs (and hence knowledge gaps) for liana‐tree competition (in particular liana quantum efficiency and stomatal regulation parameters). In the future, it can easily serve to evaluate the impact of site or treatment on liana traits or test the hypothesis of liana PFT homogeneity. Lianas indeed exhibit a broad diversity in a very wide range of processes. Yet, they were all assumed to be part of a unique and representative plant functional type. Additional observations that would feed the meta‐analysis could inform us if multiple liana functional types need to be accounted for according to their natural variability and the respective role that they have on forests.

Some specific liana parameters were systematically the largest contributors to model output uncertainty (growth respiration, quantum efficiency, plant hydraulics) and the list of these parameters largely overlaps with the ones of tree‐PFT parameters from previous uncertainty analyses. Table 4 compares the uncertainty analysis results for ecosystem NPP from this study and from Raczka et al. (2018) and Dietze et al. (2014). Except for the height allometric coefficients (not considered in previous studies and quite specific to lianas, see Figure 1), all parameters identified as critical for the liana‐tree competition were previously identified as crucial for trees as well (note that soil–plant water conductance was replaced by a set of mechanistic parameters in this study, e.g. *stoma_psi_b*, *stoma_psi_c* and *K*_max_). It would be interesting to extend the uncertainty analysis accounting for both tree and liana parameters. While it would increase the number of parameters to constrain, it would also allow refining the mechanisms behind which lianas compete more with pioneering trees.

**TABLE 4:** Comparison of most important liana parameters with the tree PFT (previous analyses of Dietze et al. (2014) and Raczka et al. (2018)). Coloured bold parameters (blue, green and grey) highlight analysis similarities even though the model structure (and hence parameter names) differ. From Raczka et al. (2018), we included analyses with (posterior_re) and without (posterior) random effects

This study ecosystem NPP (posterior)		Raczka NPP (posterior)	Raczka NPP (posterior_re)	Dietze NPP (posterior)
BCI, Panama	Paracou, French Guiana			
**Growth respiration (33%)**	**Growth respiration (44%)**	**Soil–plant water conductance (>50%)**	**Quantum efficiency (50%)**	**Growth respiration (>50%)**
**Quantum efficiency (24%)**	**stoma_psi_b (10%)**	**Growth respiration (28%)**	Leaf respiration (25%)	**Soil–plant water conductance (11%)**
**stoma_psi_b (24%)**	b1Ht (9%)	Stomatal slope (5%)	**Soil–plant water conductance (12%)**	Stomatal slope (10%)
**Kmax (7%)**	b2Bl (7%)		**Growth respiration (12%)**	**Quantum efficiency (7%**)
	b2Ht (7%)			Carbon balance mortality (6%)
	**Quantum efficiency (7%)**			

The uncertainty analysis also highlighted processes that lack a sufficiently mechanistic approach to be constrained with existing trait data, and therefore contributed the most to the overall variances. As high uncertainties were similarly found in growth respiration of other PFTs in ED2, the large relative variances of respiration parameters represent more a general feature of the ecosystem model structure than a specific characteristic of the liana PFT. Liana climbing (as represented by the allometric coefficients *b1Ht* and *b2Ht*) and plant growth respiration are oversimplified in the ED2 model and it is therefore difficult to constrain the corresponding parameters (not directly observable) otherwise than by refining the underlying processes or through parameter data assimilation. Lianas share the same model limitations as other PFTs in terms of model inadequacy to represent certain eco‐physiological processes such as growth respiration, while being further featured by growth‐form specific uncertain mechanisms (climbing). It also demonstrates how lianas in competition with trees for the same resources are sensitive to the same processes (even with very different parameterizations and in very contrasted sites).

Liana data remain much more scarce than tree data. Beyond the trait data priorities identified above, the liana plant functional type must be enriched by new datasets and additional priors as novel observational traits accumulate in order to increase liana parameter constraints and hence improve model simulation accuracy. For instance, while it seems that leaf optical properties differ between tree and liana leaves (Castro‐Esau et al., 2004; Guzmán et al., 2018), reflectance/transmittance parameters were not considered in this study. Because of the role they play in the forest radiative transfers (Viskari et al., 2019), those parameters (and others) should be included in the future.

### 4.3 Study limitations and perspectives

As a vegetation modelling study, this research has important intrinsic limitations, the most critical of which is probably its ecophysiological boundaries. Lianas and trees interact more than through resource competition. It has been demonstrated that lianas can damage their hosts directly by mechanical abrasion and passive strangulation or indirectly by increasing the hosts’ susceptibility to wind damages and likelihood of treefall (Putz, 1984). Hence, in reality, lianas might affect tree productivity and mortality rates in more ways than those that can be determined physiologically while we only focused on the latter in this study. Furthermore, many of the model predictions presented here are preliminary and should be validated using new and relevant datasets. Such a model–data fusion loop approach would help keep improving and refining model accuracy.

Similarly, liana abundance is not only driven by a combination of competition with self‐supporting plants but also by fundamental limits on their capability to exist under different abiotic conditions, for example, freezing air temperature (Ewers, 1985; Schnitzer, 2005). In addition, several putative mechanisms of increasing liana abundance in the Neotropics (Schnitzer & Bongers, 2011) were not considered at all in the vegetation model. They were either omitted because of the time‐scale of this study (e.g. elevated atmospheric CO_2_) or they could not be easily included in the vegetation model structure (e.g. hunting which might affect tree seed dispersal more than liana's). Nutrient deposition, which has been proposed as a possible long‐term explanation for the increasing liana abundance (Asner & Martin, 2014; Schnitzer & Bongers, 2011), was also not taken into account in this study because of its limited temporal scope. Despite recent evidence suggesting that nutrients do not play a major role in liana–tree interactions (Pasquini et al., 2015; Schnitzer et al., 2020), such a mechanism could still be considered in future (longer term) studies. Recent ED2 model developments (Levy‐Varon et al., 2019; Medvigy et al., 2019) enable extending competition factors to nutrient acquisition and disentangling of water and nutrient below‐ground competition although empirical evidence does not currently support the need to include nutrients as a strong driver of liana population growth.

In addition, there are several plant processes that could be taken into account in the future for a more accurate representation of lianas in ED2. Currently, the model assumes an infinite ability for xylem refilling for both lianas and trees, while lianas, in some cases, may be better than trees at cavitation recovery (Ewers & Fisher, 1991; Fisher et al., 1997). As lianas cavitated more during our simulations, they also refilled their cavitated vessels more than trees. Yet, this could be simulated more explicitly in the future. Differences in leaf phenology were also not accounted for while lianas produce leaves over a greater fraction of the year than trees whatever their successional status (Putz & Windsor, 1987). Actually, all plants were simulated as evergreen in our model runs while few lianas and nearly half of the canopy trees on BCI are brevi‐ or facultatively deciduous and hence lose a fraction of their leaves during parts of the dry season (Putz, 1990). In the future, contrasting seasonal phenology strategies should be considered to reproduce the seasonal differences in liana and tree growth (Schnitzer, 2005).

It must also be emphasized that the positive impacts of liana removal on forest productivity and carbon sequestration as observed in experimental plots (van der Heijden et al., 2015) and confirmed in our model simulations might be temporary. The substantial benefit of tree growth after liana cutting (Mills et al., 2019) presumably diminish with time, even if some seminal studies suggest that they could persist as long as 6–10 years after removal (Kainer, 2014; van der Heijden et al., 2019). Longer model simulations validated on larger experimental datasets should allow the quantification of those long‐ versus short‐term liana‐removal impacts.

Finally, the choice of the sensitivity analysis (parameters were varied one‐at‐a‐time) makes it dependent on the default model parameter choice. Yet, the default model parameterization in this study was either set up by the median of the prior distribution (and hence determined by expert opinion) or by the meta‐analysis (and therefore reflects both the a priori knowledge and measurements of the traits). Consequently, the sensitivity analysis takes into account the most likely value of a given parameter and its variability in the presence or absence of observational data. However, other global sensitivity tools could additionally inform us about model uncertainty and the sources of competition between lianas and trees.

## 5 CONCLUSIONS

We presented in this study the first vegetation model able to disentangle the contribution of water and light in the competition for resources between lianas and trees. While being critical for the fundamental understanding of forest dynamics, it is a question that is extremely difficult to answer as isolating below‐ and above‐ground competition between lianas and trees requires heavy manipulations and measurements. Vegetation models therefore have an important role to play to unravel interactions between plant functional types. By further developing a liana PFT in the ecosystem demography model (ED2), and analysing it with the bioinformatics toolbox PEcAn, we identified that liana quantum efficiency and stomatal regulation parameters were the most critical parameters controlling liana productivity and hence the liana versus tree competition. Model simulations with parameters constrained by data successfully reproduced the magnitude and seasonality of GPP and ET, and the magnitude of aggregated properties such as LAI and AGB. Competition with lianas was predicted to negatively impact tree growth (between −30% and −40%) and reduce forest net productivity in both sites. Uncertainty analyses suggested that water competition was more critical in the relationship between lianas and trees than expected. Indeed, water acquisition dominated the yearly growth‐form competition on BCI and was even important in a relatively wet site as Paracou. This workflow can now serve to predict the impacts of lianas on tropical forest carbon sink strength or storage at large scale or in a climate change context where decreased rainfall, increased disturbance and stronger seasonality are expected to promote lianas.

## AUTHORS' CONTRIBUTIONS

F.M., M.Di. and H.V. designed the study; F.M. implemented the model, ran the simulations and processed the results with inputs and support from M.Di. and B.C. for PEcAn technical aspects; F.M., H.V., B.C., S.A.S., C.M.S‐M., J.S.P., X.X., M.S., H.P.T.D.D., M.De., D.B., M.L., L.S.S., & M.Di. contributed to the interpretation of the results, participated in manuscript writing and critically revised the study; In addition, numerous co‐authors contributed to collect data used in this study: X.X., J.S.P. and C.M.S.‐M. (rooting depth allometric data), M.S. (stomatal slope), D.B. (Paracou fluxtower data) and M.De. (BCI fluxtower data).

### PEER REVIEW

The peer review history for this article is available at https://publons.com/publon/10.1111/1365‐2745.13540.

## Supporting information

Supplementary Material

## Data Availability

All data necessary to reproduce the results presented in this study, including ED2 source code, PEcAn source code, initial vegetation conditions, MET drivers and parameter distributions and traits, can be found on https://zenodo.org/record/4115206#.X5AyjHUzYig (https://doi.org/10.5281/zenodo.4115206) (Meunier et al., 2020).
