# Supplementary material for: Unraveling the relative role of light and water competition between lianas and trees in tropical forests: A vegetation model analysis
Source: J Ecol. 2020 Nov 29;109(1):519–40. doi: 10.1111/1365-2745.13540 (PMC7839527; doi:10.1111/1365-2745.13540)
Supplement: Supplementary file 1 — Supplementary Material [file JEC-109-519-s001.docx]

Supplementary material

# Appendix A: ED2 processes explored in this study

In ED2, PFT carbon pools and sizes are derived from allometric equations. In particular, woody biomass (B_d_), and leaf biomass (B_l_) are scaled with the cohort DBH while the maximum rooting depth (R_d_) is related to the cohort height:


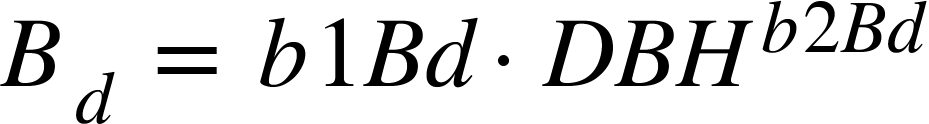


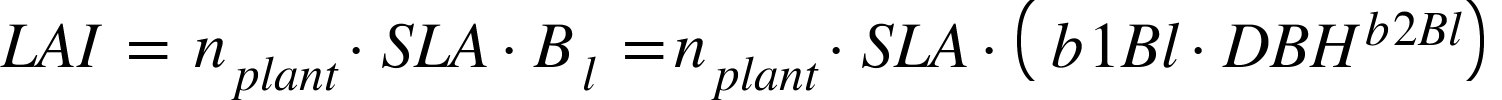


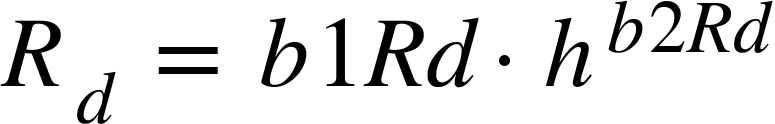


where b1Bd (resp. b1Bl/b1Rd) and b2Bd (resp. b2Bl/b2Rd) are the intercept and the slope of the woody biomass (resp. leaf/rooting depth) allometric equation. Cohort LAI is directly derived from the leaf biomass using the specific leaf area (SLA) and the plant density (n_plant_).

The maximum rooting depth is further used to partition the root biomass (B_r_) in the different soil layers using a cumulative exponential function and a shape parameter β. Cohorts extract water from each soil layer i (whose upper and lower depths are z_1,i_ and z_2,i_, respectively) through their total root area in this layer Root Area_i_, derived from the root biomass fraction in this layer and the specific root area (SRA):


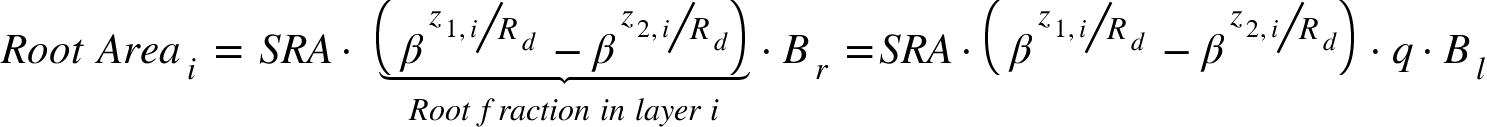


In the previous equation, we also see how the root biomass is derived from the leaf biomass thanks to a scaling coefficient q: $B_{r} =q.B_{l}$.

Cavitation is considered through a reduction of the stem conductivity (K) as compared to its saturated conductivity (K_max_) with decreasing wood water potential (Ѱ_wood_):


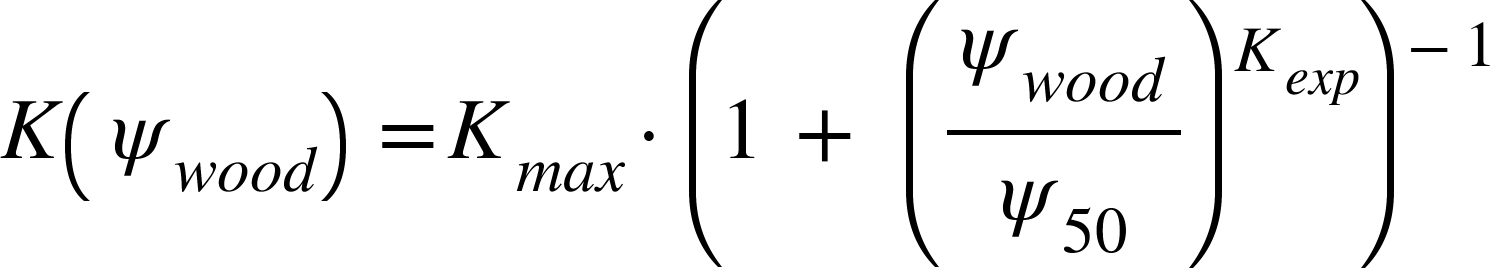


The loss of conductivity is parameterized using the potential at which 50% of the conductivity is lost (Ѱ_50_) and a shape coefficient (K_exp_). The plant water status (Ѱ_leaf_) further affects the plant photosynthesis through a reduction factor f_sw_ (comprised between 0 and 1) using two empirical stomatal closure curve parameters, stoma_psi_b and stoma_psi_c:


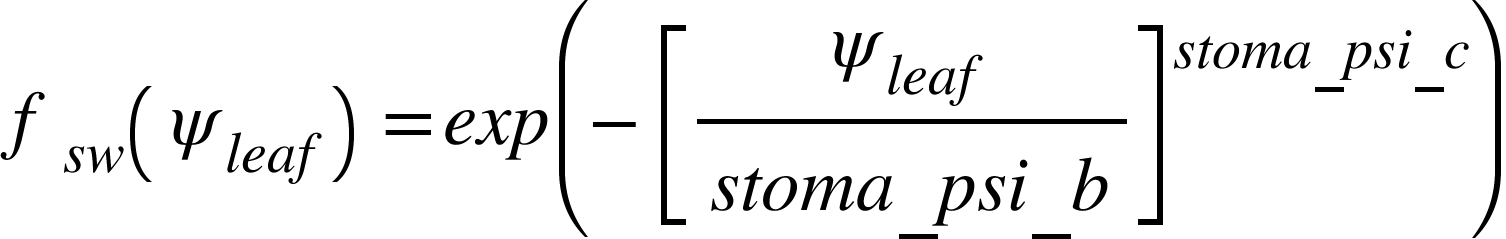


Leaf-level photosynthesis (A) is determined by the minimum of its light-limited rate (J_e_) and its Rubisco-limited (J_c_) rates when the stomata are open:


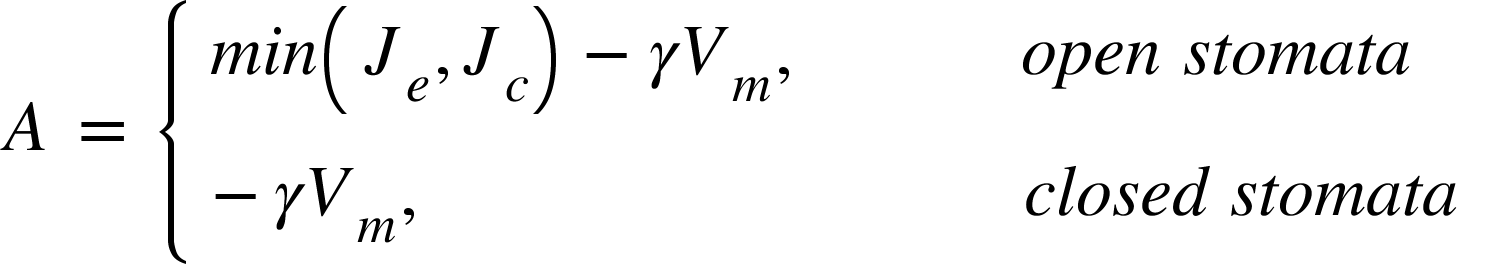


where ɣV_m_ represents the leaf dark respiration. ɣ is a dark respiration factor and V_m_ denotes the capacity of Rubisco to perform the carboxylase function, determined by the maximum photosynthesis capacity (V_m0_) and a temperature (T) function:


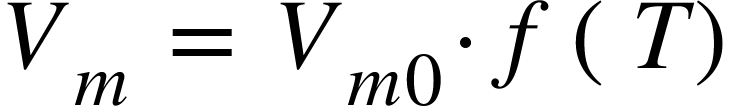


The light-limited rate of photosynthesis increases with photosynthetically active radiation (PAR):


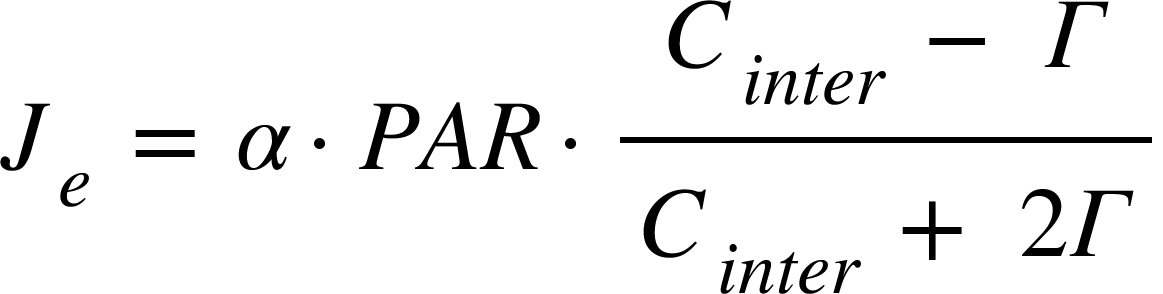


with α the quantum efficient, 𝛤 the compensation point for gross photosynthesis and C_inter_ is the intercellular concentration of CO_2_.

The Rubisco-limited CO_2_ rate of photosynthesis is determined by:


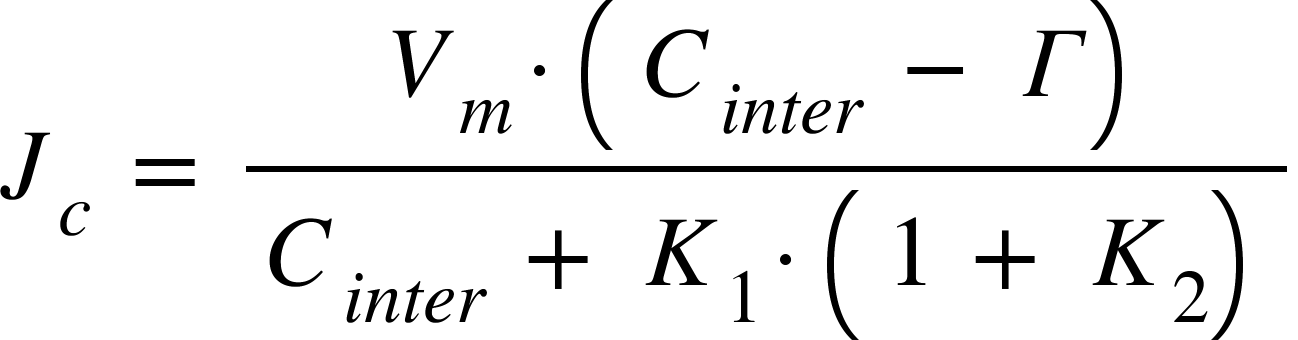


with K_1_ and K_2_ temperature-dependent coefficients. Photosynthesis and transpiration are coupled through the stomatal conductance for water (g_sw_):


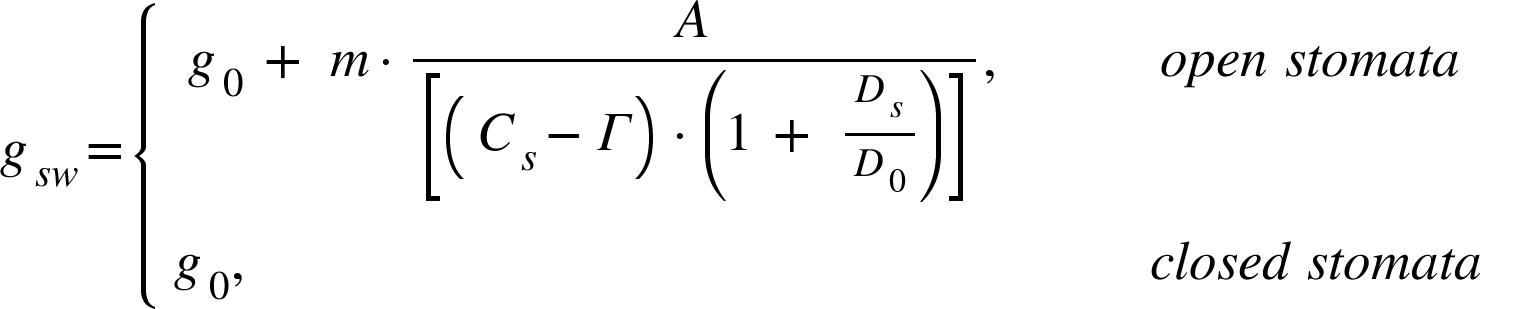


where m is the stomatal slope, D_0_ another empirical constant and g_0_ the cuticular conductance. C_s_ is the concentration of CO_2_ in the boundary layer.

Plant mortality is determined by the sum of the density-dependent (mort^DD^) and the density-independent (mort^DI^) mortality rates. The former declines exponentially with the ratio of the cohort carbon balance (CB) compared to the carbon balance of a cohort that would experiment no stress (CB*); the latter is the sum of a PFT-independent tree fall disturbance rate (λ_TF_) and a PFT-specific rate (mort_3_):


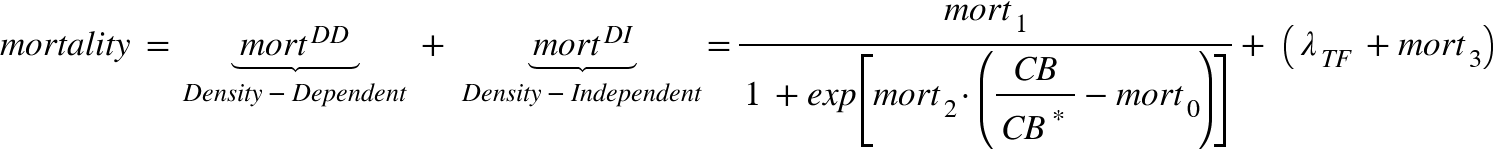


where mort_0_, mort_1_ and mort_2_ are the three shape parameters for the density-dependent mortality rate, and mort_3_ is a density-independent parameter that represents the ageing mortality of each PFT.

In addition, we also considered the impact of the following PFT-specific parameters:

- the leaf and root turnover rates that determine the leaf and root maintenance costs (proportional to the corresponding biomasses);
- the root and growth respiration that reduces the cohort-scale carbon gain as a tax on GPP once added to the leaf respiration;
- the plant wood density;
- the leaf turgor loss point that influences leaf phenology.

# Appendix B: Liana height limitation

In ED2, tropical tree cohort height (h_tree_) is derived from DBH through:

|  | 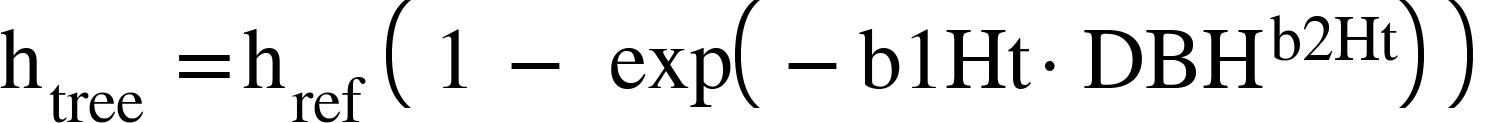 | Eq. B1 |
| --- | --- | --- |

where h_ref_, b1Ht and b2Ht are PFT-specific empirical parameters whose default parameterization comes from Poorter et al. (2006). Liana cohorts were allowed to follow a unique height allometry implemented with a DBH-offset (ΔDBH), as compared to the default tree equation:

|  | 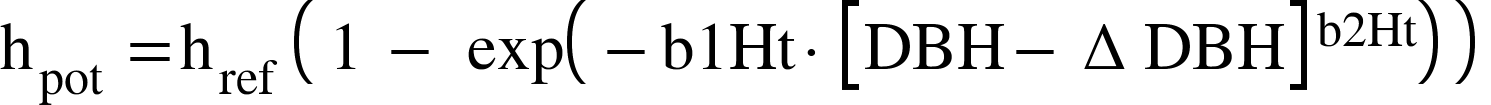 | Eq. B2 |
| --- | --- | --- |

where h_pot_ represents the maximum potential height for a liana cohort with a specific DBH and h_ref_, b1Ht and b2Ht are the liana-specific shape parameters whose prior parameterization was chosen to distribute plants with small DBH in the canopy. Liana DBH-offset is a cohort property that can be positive or negative depending on the realized height of the liana cohort as compared to its predefined one (Fig. 1). Positive and negative values respectively represent liana cohorts shorter or taller than their predicted height given by Equation B1 (parameterized for liana).

Liana cohort height (h_liana_) was then limited by the height of the tallest tree within each patch incremented by a small offset (h_offset_). This height restriction prevents lianas from unrealistically growing taller than other plants in their close vicinity:

|  | 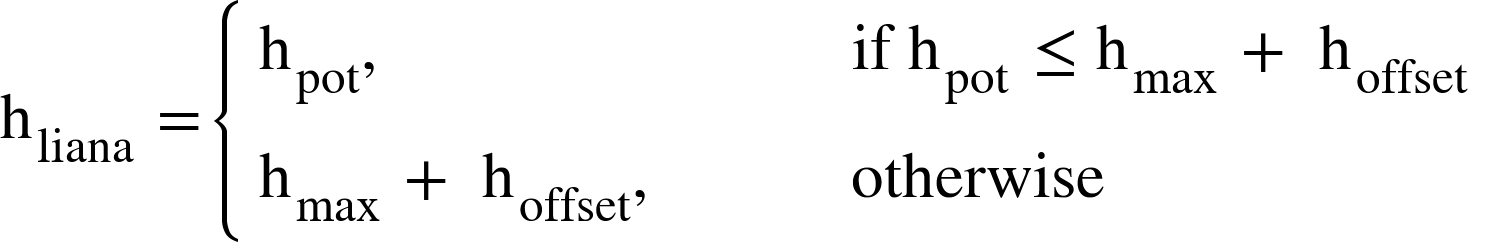 | Eq. B3 |
| --- | --- | --- |

where h_max_ is the height of the tallest tree cohort in the patch and h_pot_ is computed using Equation B2. Therefore, as opposed to trees, liana growth is a two-step process: the available carbon is first allocated to allow the liana to grow in diameter and further calculate a corresponding potential height (Equation B2), which is then restricted, if necessary, by the size of the tallest tree within that patch (Equation B3).

For liana initial height distribution, a maximum height was first calculated using Equation B1 (with the corresponding liana parameters) and then reduced to the height of the tallest tree in the vicinity if exceeding it. Initial liana ΔDBH was calculated as the difference between the individual’s actual DBH and the DBH that the cohort should have to reach a given height:

|  | 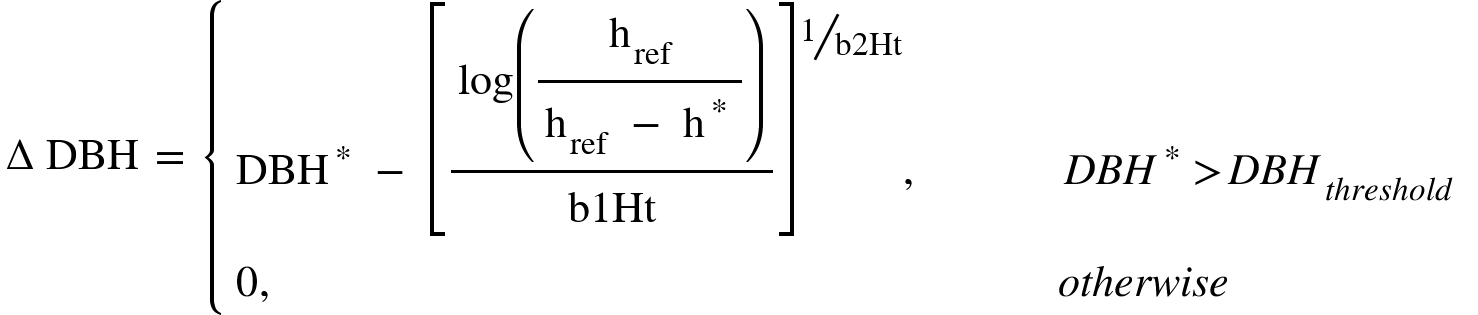 | Eq. B4 |
| --- | --- | --- |

with h* and DBH* the liana individual initial height and DBH, respectively. Liana initial height was restricted for all individuals larger than a threshold DBH fixed to 3cm: all lianas larger than 3cm overtop the tallest tree in the vicinity (whose height varies between 15 and 35m on BCI, see Figure 1).

# Appendix C: The liana plant functional type in ED2

The uncertainty analysis presented in this study heavily leans on the liana PFT introduced in an earlier publication [(di Porcia e Brugnera et al., 2019)](https://www.zotero.org/google-docs/?EEO7lF). Yet, some new features had to be implemented and others modified for the sake of this very research. Most important aspects of the liana PFT specific functioning as well as the differences with the original version are given below.

In ED2, lianas are simply another plant functional type similar to free-standing trees except for a few processes specific to climbers and their default parameterization. First, and most importantly, liana height is limited by the size of neighbouring trees. In the original implementation, liana cohorts were tracking the same tree cohorts from their establishment to their death. Those tracking lianas could only overtop their host by a small offset even if the liana-supporting tree was not the tallest plant in the patch. We modified this process in this study for two reasons. First, we wanted to make the lianas able to reach the top of the canopy even when their original supporting tree did not, *i.e.*, accounting for host changes. Second, when starting from prescribed initial conditions as we did (inventories), associating every liana with a unique host requires strong assumptions, which we did not want to make. Therefore in the new version, liana height limitation is patch-based rather than cohort-based: lianas can only overtop the tallest tree within a patch by a small offset, as fully described in Appendix B.

As a consequence of such a change, we also dropped the direct impact of liana on its supporting tree. In the paper of di Porcia e Brugnera et al. (2019), increased turnover rates were applied to colonized plants to account for increased investments in acquisitive tissues. The direct light competition was now assumed to be entirely simulated by the radiative transfer module of ED2: climbing lianas indeed shade their host (and all plants below their vertical position), which directly impacts their photosynthetic activity. The cohort fusion-fission criteria of the parasite-host relationship were also released. Instead cohort fusion was only allowed when lianas cohorts shared both similar sizes (DBH) and heights.

The vegetative reproduction mechanism of lianas was kept identical to the original implementation: the excess carbon, after all maintenance and respiration processes are computed, is primarily allocated to reproduction (81%) rather than to growth (19%). A large fraction of the reproduction NPP (95%) is also sent to the litter pool due to reproduction mortality, which mimics large carbon investments for seeds and new shoots.

We also kept as is the ability of lianas to survive treefall disturbance events. The model assumes that, when a disturbance occurs, only a limited fraction of small plants (< 10 m) survive. Yet, it has been demonstrated that lianas often survive in gaps after the fall of their host (Putz, 1984). To simulate this, we assume a higher probability of survival to treefall events for large lianas (80%). The fallen lianas lose part of their biomass to represent the damage: they are forced back to the predefined allometries and all other tissues are rescaled accordingly. Lost biomass is then transferred to the relevant soil carbon pools.

Finally, a large effort in our original publication was dedicated to define liana-specific allometries and choose an appropriate parametrization for the liana PFT. This effort was continued in this research in updating the existing observational data to feed the analysis. Liana-specific allometries were kept unchanged as compared to the original formulation, unless otherwise specified (Tables 1 and 2). The comprehensive liana allometries are listed in the supplementary section 1 of di Porcia e Brugnera et al. (2019).

# Appendix D: PEcAn Meta-analysis

PEcAn details each PFT as a list of model parameters, their associated prior probability distributions, and an ensemble of plant species, stored in PEcAn’s companion database, BETYdb. Posterior distributions are estimated from the available trait information (mapped to model parameters and plant species composing the PFT) using a linear mixed model:

|  | 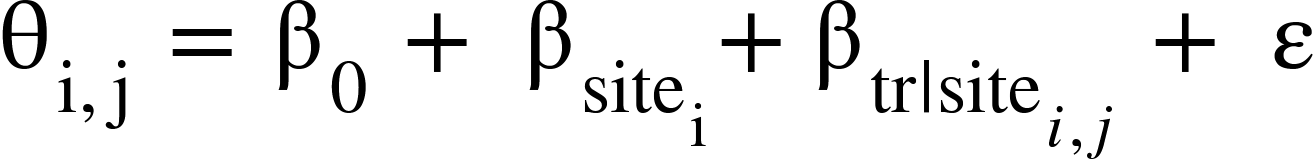 | Eq. D1 |
| --- | --- | --- |

in which the true trait mean of the p^th^ parameter $\theta_{i,j}$at the i^th^ site and j^th^ treatment is a combination of the global trait mean ($\beta_{0}$), a Normal random effect for the site ($\beta_{{site}_{i}}$), a nested Normal random effect for the experiment treatment ($\beta_{tr|{site}_{i,j}}$), and a Normal residual variance (ε). The fitting procedure was adapted from LeBauer et al. (2013) using field data only (rejecting greenhouse experiments). In practice, less than 5% of the trait data considered involved experimental treatments, so the primary focus here was on estimating the overall mean and the within- and across-site variability. Equation C1 was fitted through PEcAn with JAGS [(Plummer, 2010)](https://www.zotero.org/google-docs/?jSXS9l) using four Markov Chain Monte Carlo chains per parameter. The estimated distribution of $\beta_{0}$was used as the trait posterior distribution for performing the ensemble and sensitivity analyses of ED2.

# Appendix E: Site supplementary information


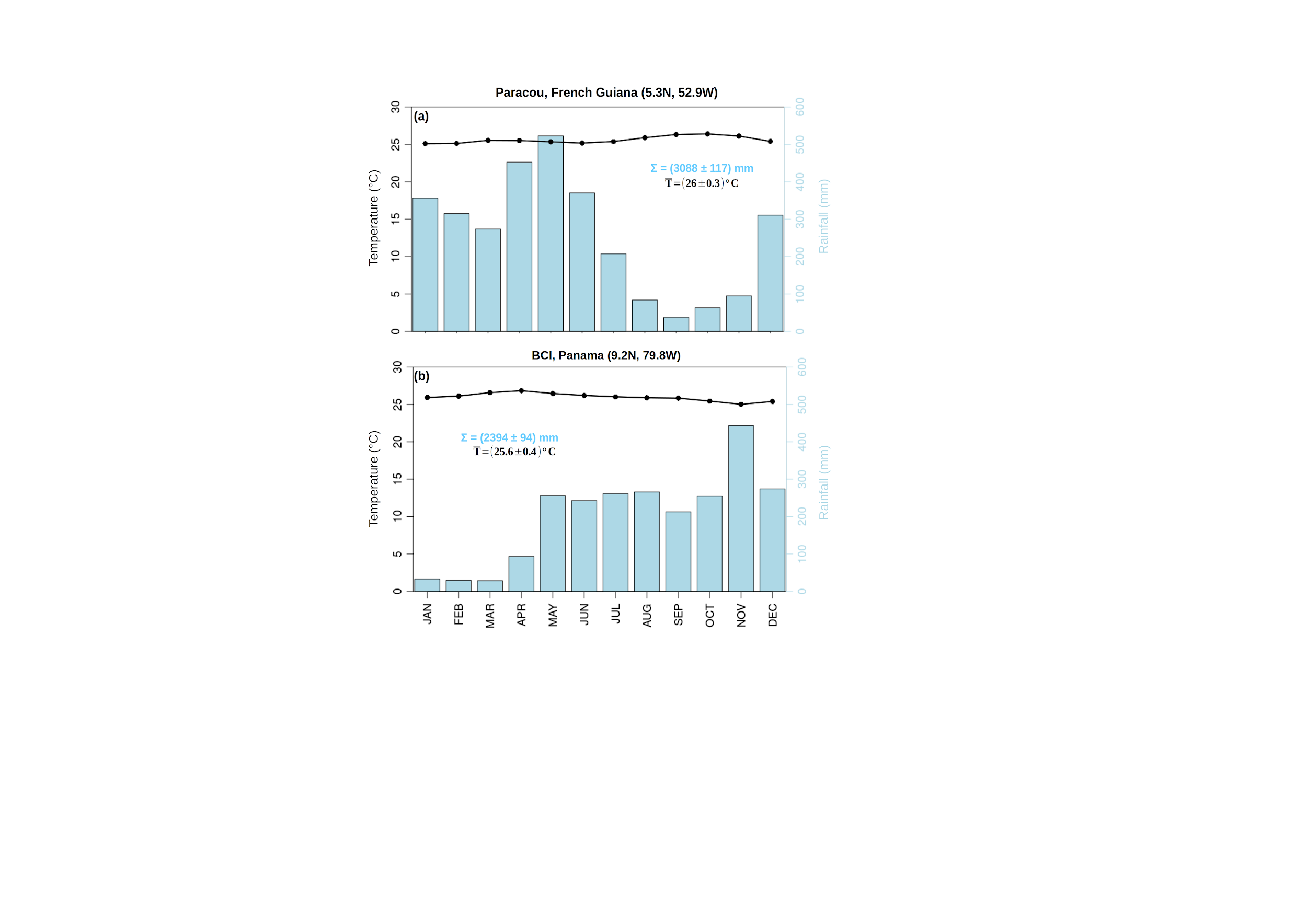


##### Figure E1: Ombrothermic graphs of the two simulated sites: Paracou, French Guiana (a) and BCI, Panama (b). Adapted from [(di Porcia e Brugnera et al., 2019)](https://www.zotero.org/google-docs/?98I7XW).

#####
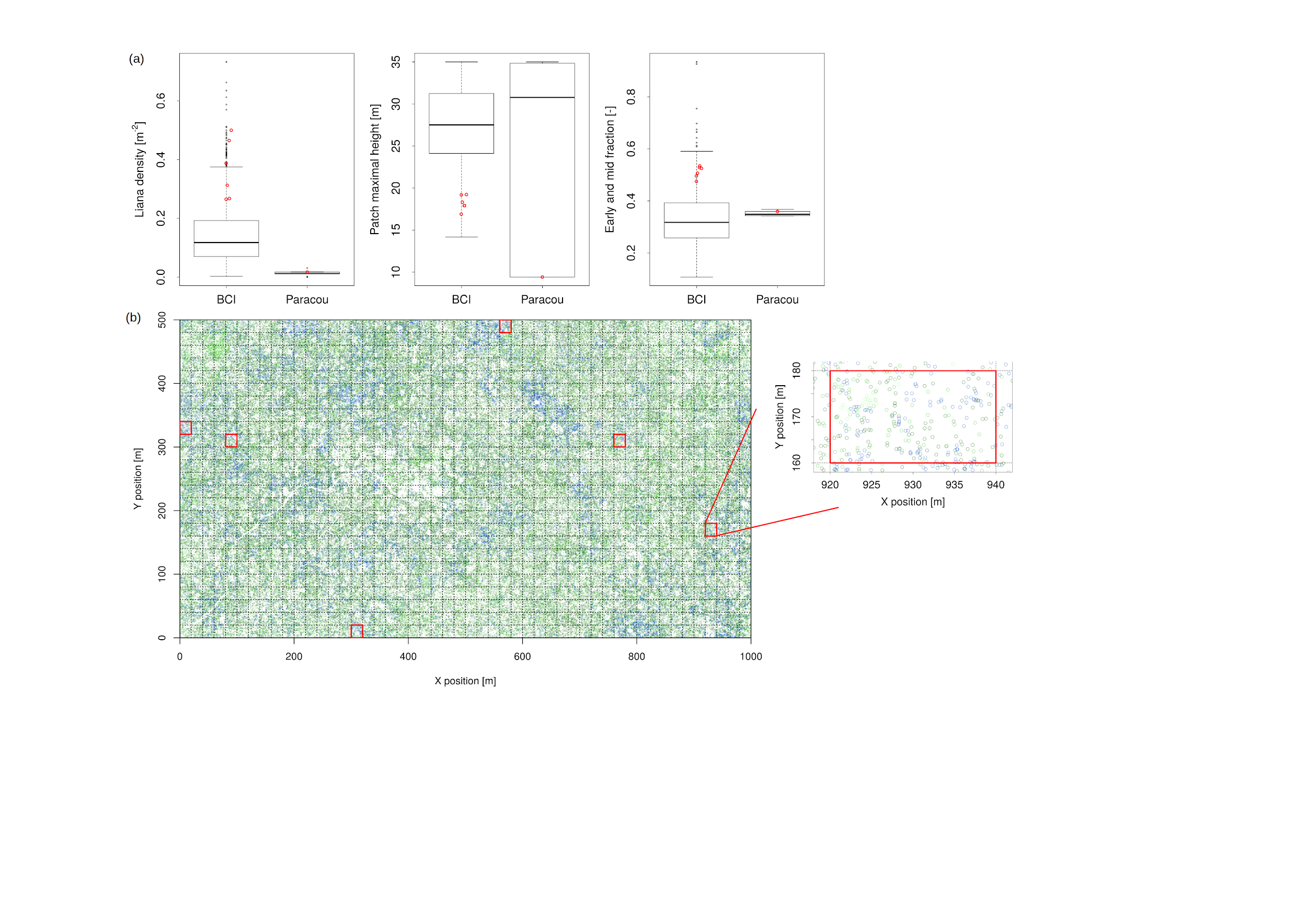


##### Figure E2: Simulated forest composition and structure on BCI, Panama and Paracou, French Guiana (panel a) and observed liana (blue) and tree (shades of green = different tree PFT) inventories on BCI, Panama alongside with the forest decomposition in young patch area (panel b). In subplot a, the forest composition and structure are represented by the liana density (left), the canopy height (middle) and the relative fraction of early and mid successional trees (right) distributions. The patches selected for simulating the young patches area in the analysis are highlighted in red in both subplots.

# Appendix F: Supplementary results


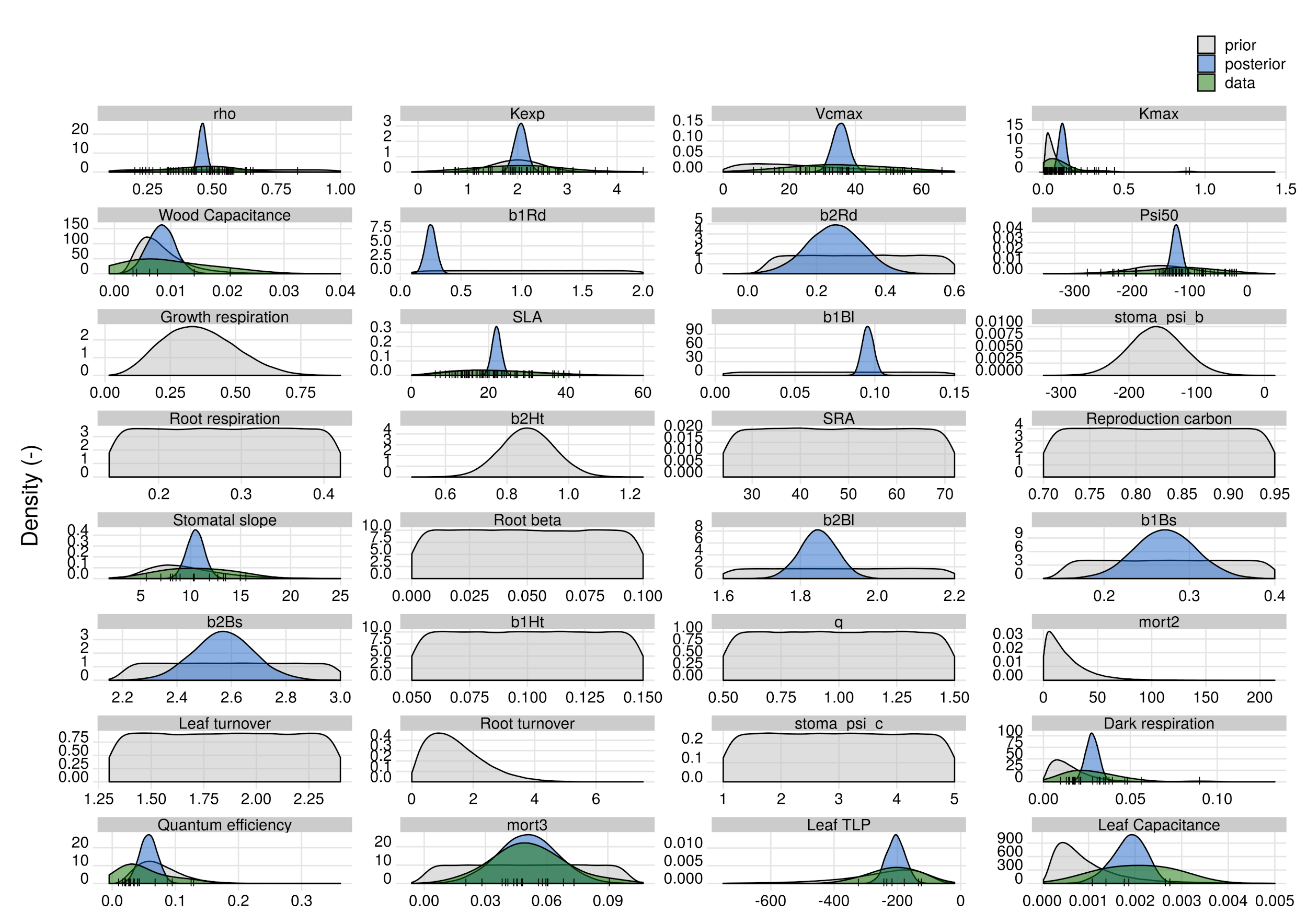


##### Figure F1: All liana PFT parameter distributions. The prior distributions (grey) are relatively broad and were defined to encompass parameter natural variability and cover all field observations (black vertical lines smoothed into the green distributions). A Bayesian meta-analysis was performed to combine the prior distributions with trait data (whenever available) to create posterior distributions (blue), which were further used to estimate both the model and parameter uncertainties. The units of each parameter are given in Table 1.

#
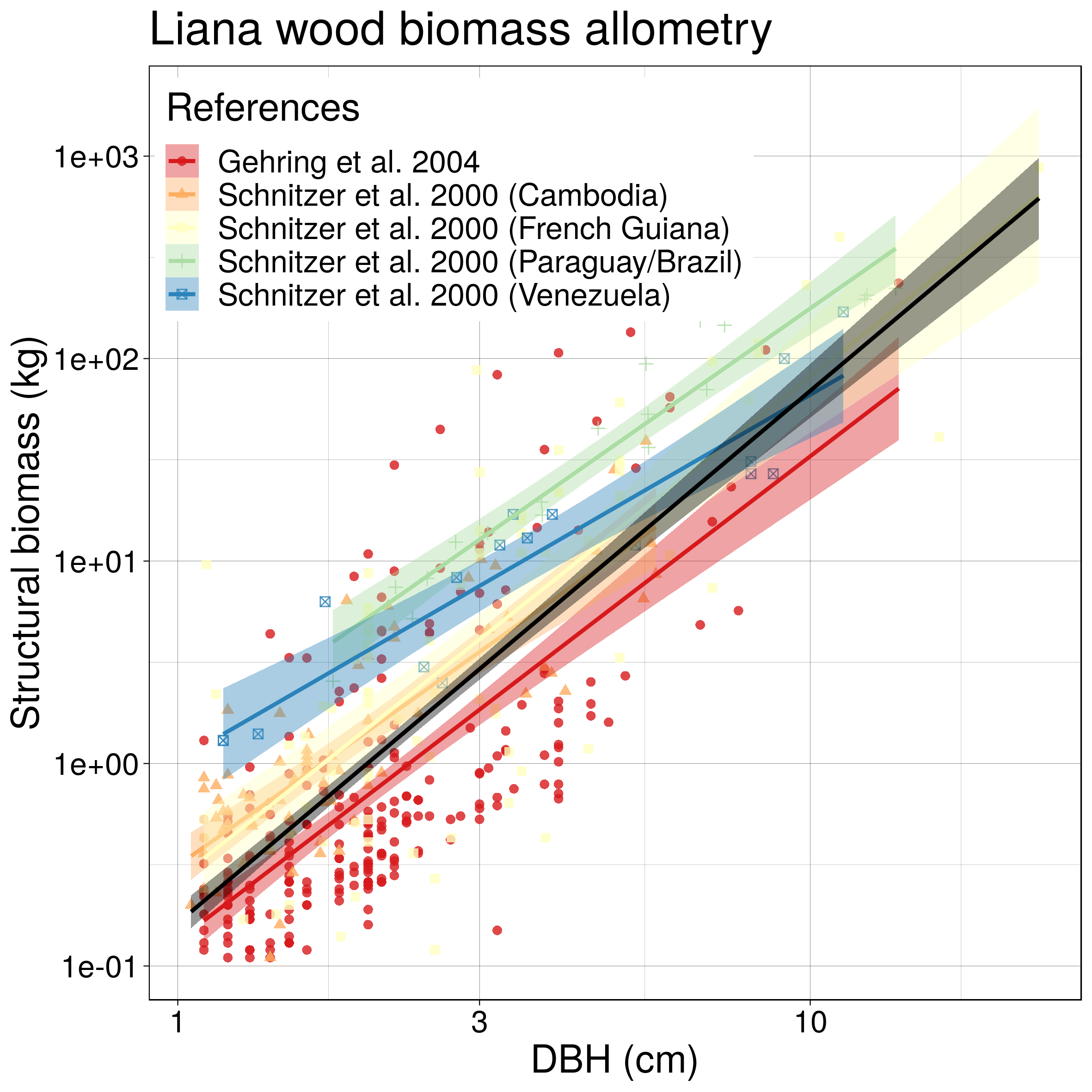


#
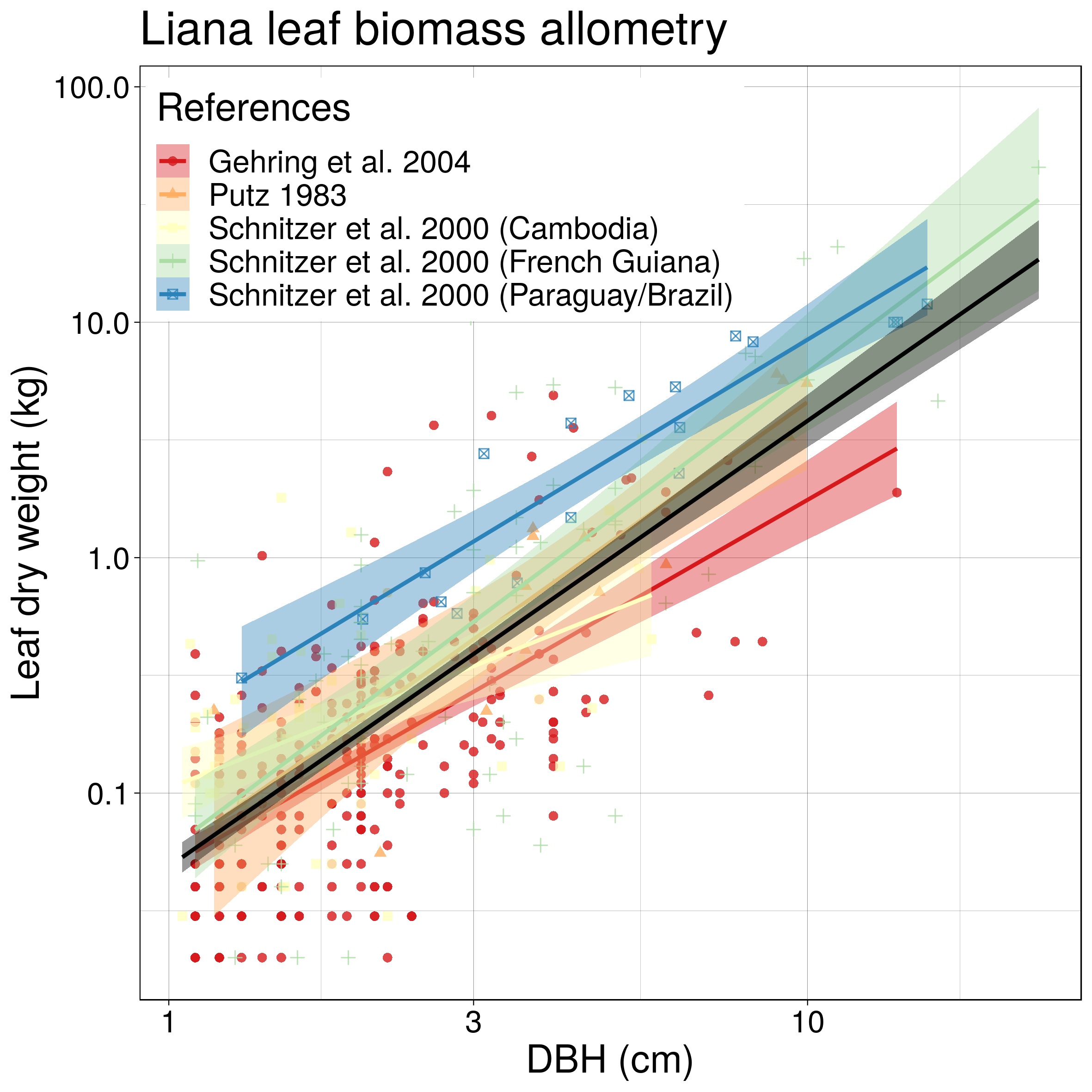


#
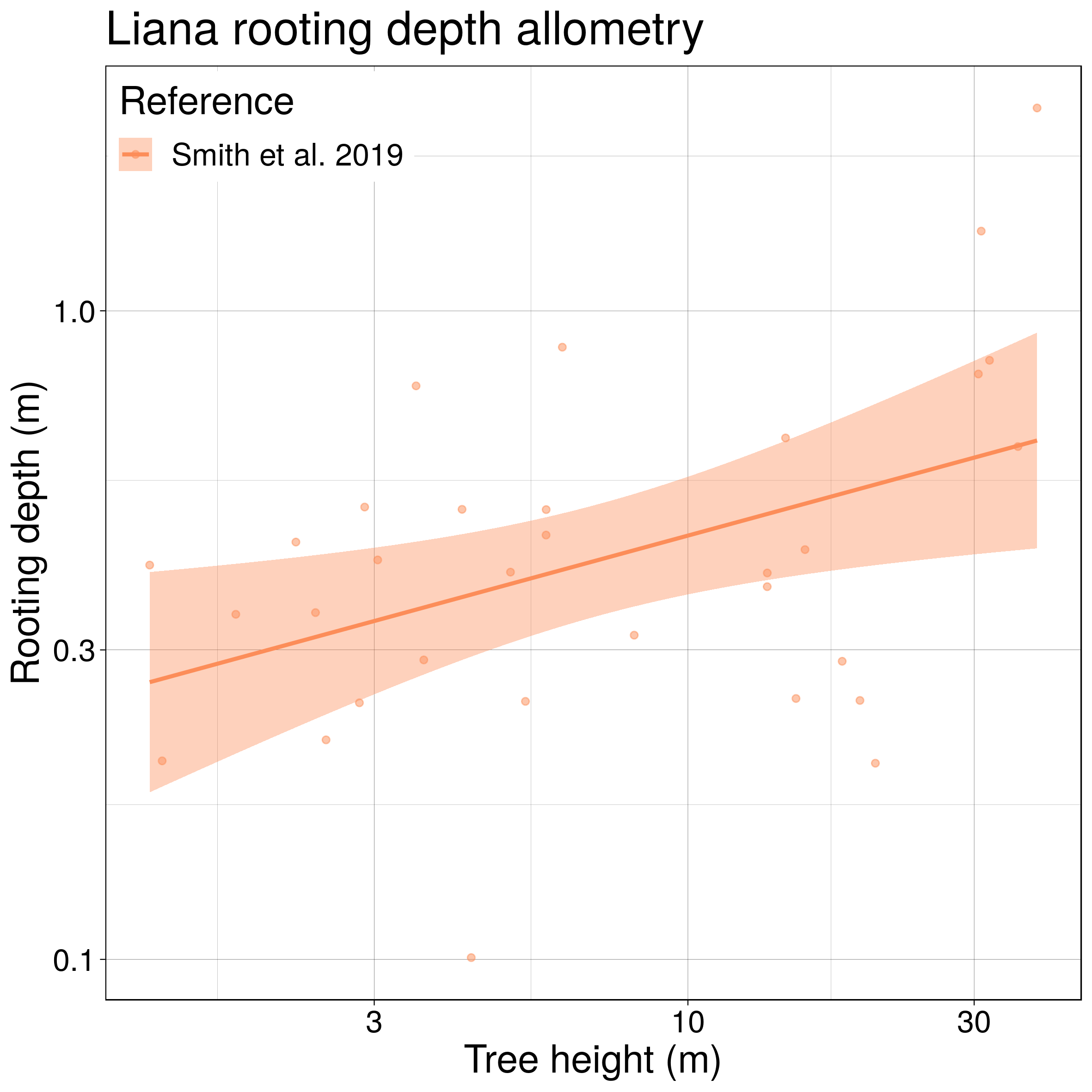


#####

##### Figure F2: Liana leaf biomass (a), wood biomass (b) and rooting depth (c) allometry as observed by different references (shapes and shades of blue). In subplots a and b, the black envelope is the linear model obtained when using all the data together.

#####

#####

###
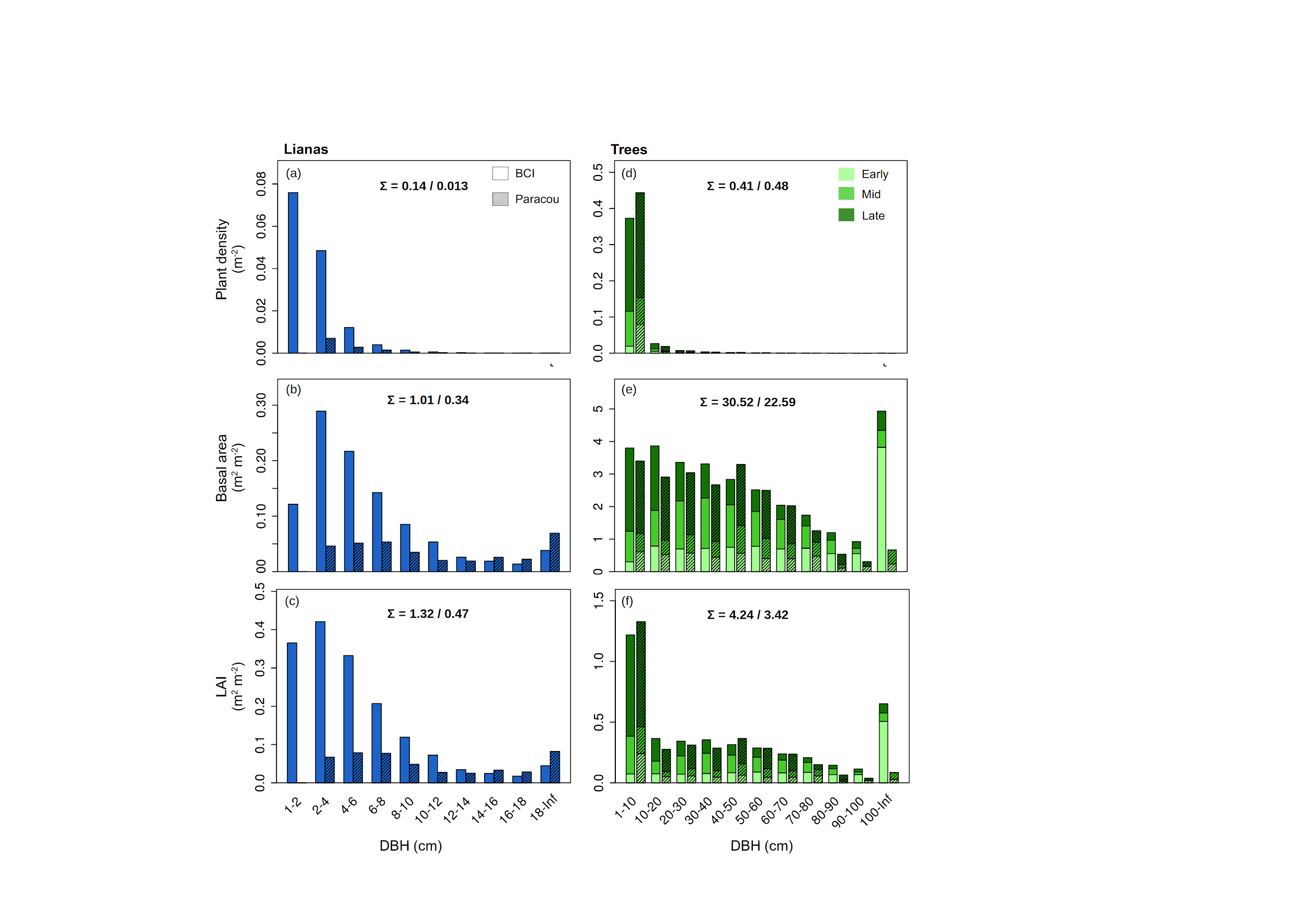


##### Figure F3: Initial liana (a-b-c) and tree (d-e-f) initial composition in terms of plant density (a-c), basal area (b-e) and Leaf Area Index (c-f) size-distributions. Plant density and basal are directly derived from local forest inventories while LAI are derived from allometric equations. The distributions are shown for both BCI, Panama (no hatching) and Paracou, French Guiana (black hatching). In addition, the tree distributions are stacked for the different tree PFTs (shades of green). In each subplot, the total (∑) is indicated for BCI (left) and Paracou (right). Small trees (1-10 cm) were generated by extrapolation from the loglog relation of size classes vs plant density.

#####


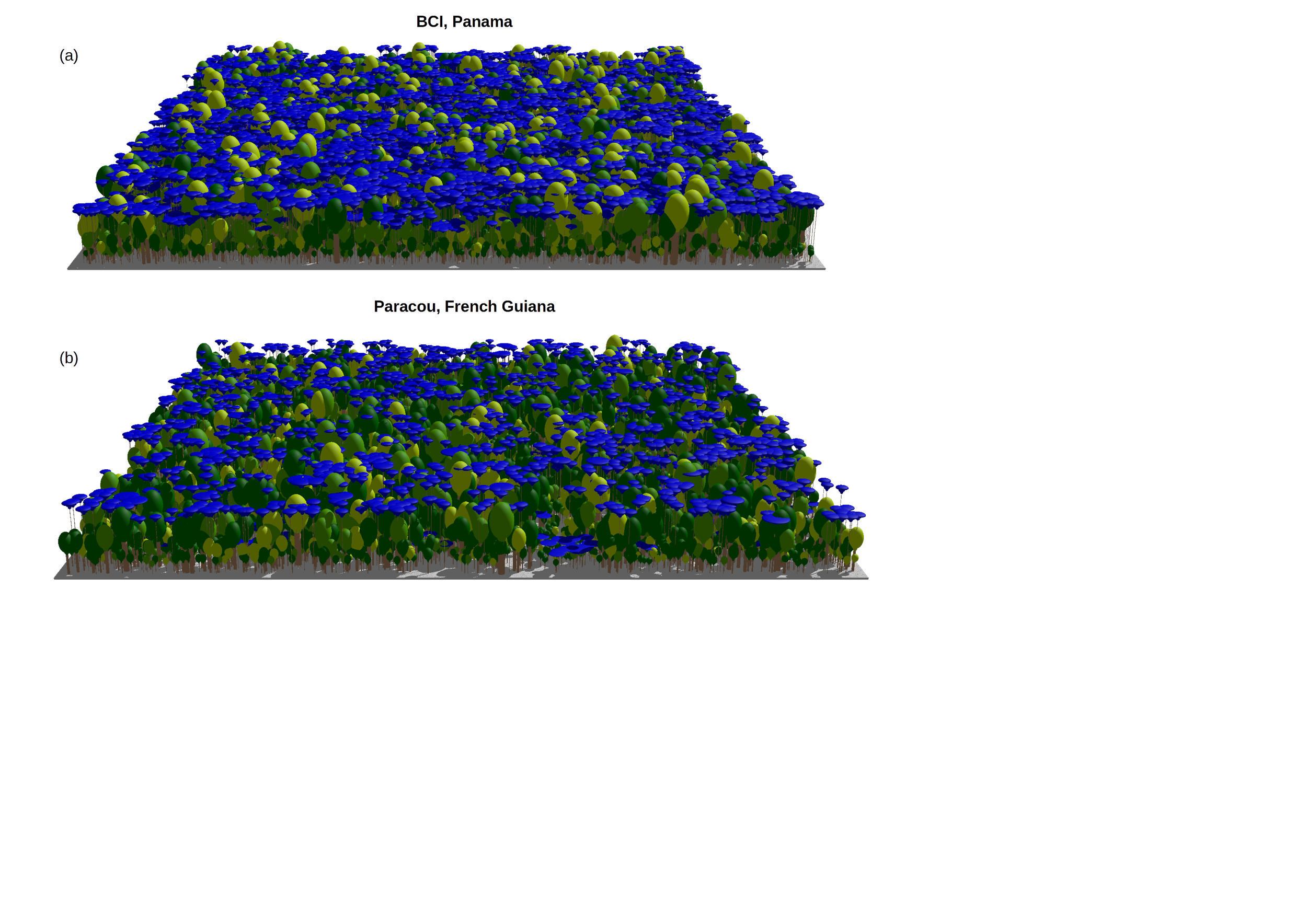


##### Figure F4: Liana (blue) and trees (shades of green = different tree PFTs) initial composition on BCI, Panama (panel a) and Paracou, French Guiana (panel b). Both panels illustrate a representative area of forest of 1 ha. To visualize the forest composition, the forest is decomposed into patches according to their simulated relative area and the liana/three cohort density and demography are preserved.

#####
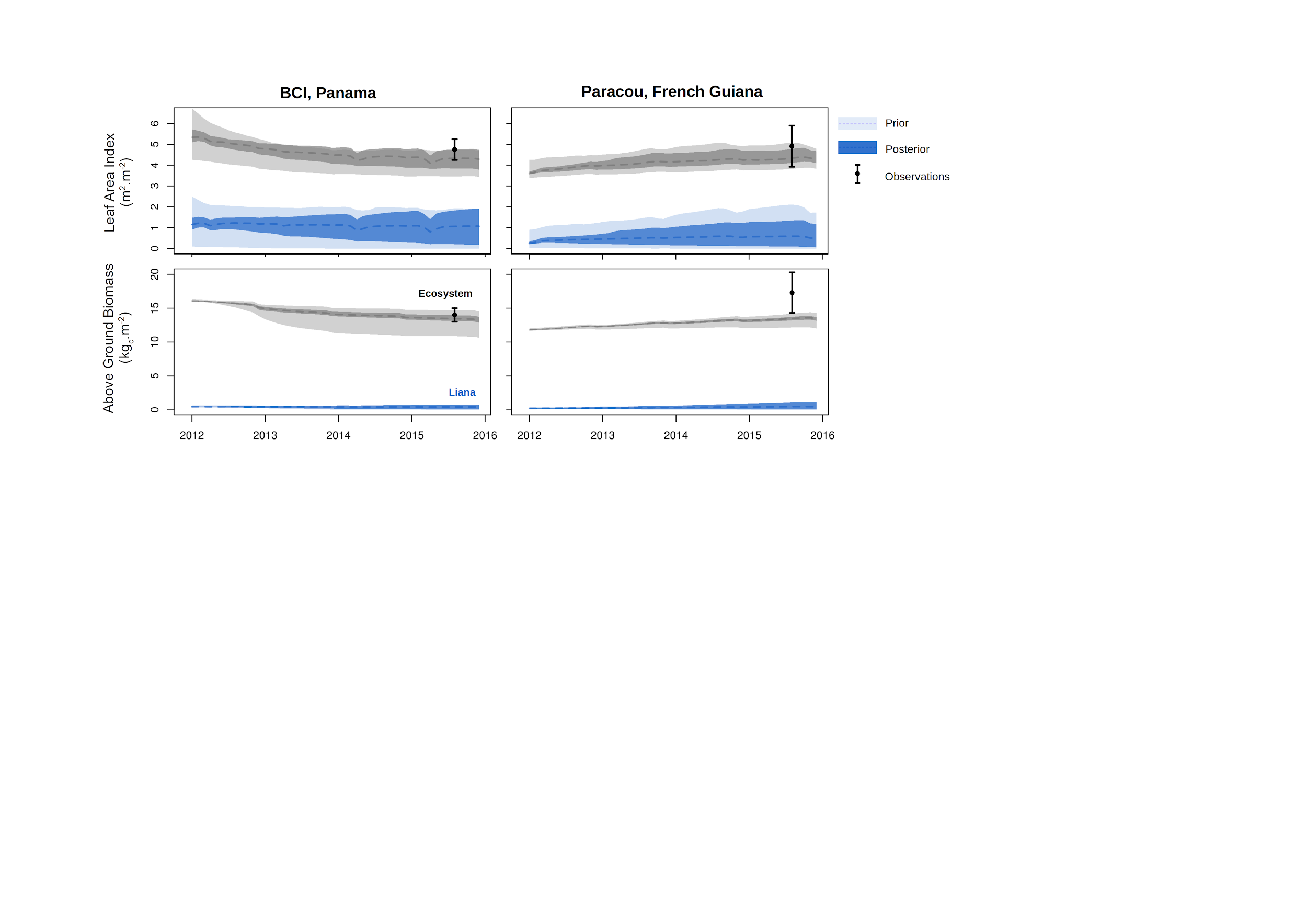


##### Figure F5: Leaf Area Index (a-b) and Above Ground biomass (c-d) in both BCI, Panama (a-c) and Paracou, French Guiana (b-d) time series as simulated by ED2. The envelopes represent the spread in the simulation at the ecosystem level (grey) or for the liana PFT (blue) when using either the prior (light) or the posterior (dark) parameter distribution.


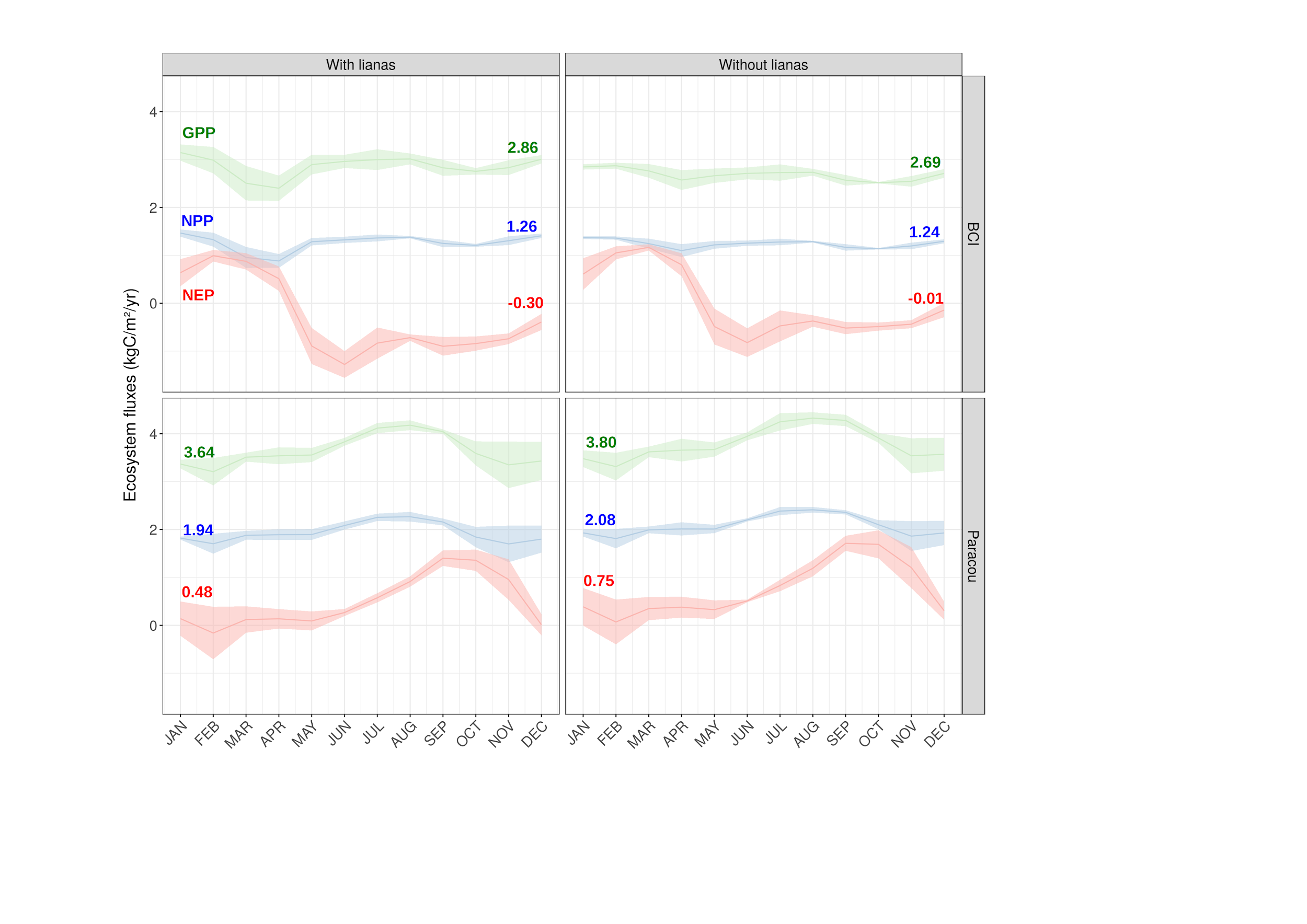


##### Figure F6: Ecosystem fluxes (green, = GPP, blue = NPP, red = NEP) on BCI, Panama and Paracou, French Guiana when switching on and off the liana PFT. The numbers indicate the yearly means (2004-2009) of the respective fluxes. In the simulations with lianas, the ecosystem fluxes are summed up over the three tropical tree PFTs and the liana PFT.
